# The Radically Embodied Conscious Cybernetic Bayesian Brain: From Free Energy to Free Will and Back Again

**DOI:** 10.3390/e23060783

**Published:** 2021-06-20

**Authors:** Adam Safron

**Affiliations:** 1 Center for Psychedelic and Consciousness Research, Johns Hopkins University School of Medicine, Baltimore, MD 21218, USA; asafron@gmail.com; 2 Kinsey Institute, Indiana University, Bloomington, IN 47405, USA; 3 Cognitive Science Program, Indiana University, Bloomington, IN 47405, USA

**Keywords:** Free Energy Principle, active inference, Bayesian brain, generative models, cybernetics, embodiment, enactivism, cognitivism, representations, consciousness, free will, mental causation, cognitive-affective development, emotions, feelings, readiness potentials, intentionality, agency, intelligence

## Abstract

Drawing from both enactivist and cognitivist perspectives on mind, I propose that explaining teleological phenomena may require reappraising both “Cartesian theaters” and mental homunculi in terms of embodied self-models (ESMs), understood as body maps with agentic properties, functioning as predictive-memory systems and cybernetic controllers. Quasi-homuncular ESMs are suggested to constitute a major organizing principle for neural architectures due to their initial and ongoing significance for solutions to inference problems in cognitive (and affective) development. Embodied experiences provide foundational lessons in learning curriculums in which agents explore increasingly challenging problem spaces, so answering an unresolved question in Bayesian cognitive science: what are biologically plausible mechanisms for equipping learners with sufficiently powerful inductive biases to adequately constrain inference spaces? Drawing on models from neurophysiology, psychology, and developmental robotics, I describe how embodiment provides fundamental sources of empirical priors (as reliably learnable posterior expectations). If ESMs play this kind of foundational role in cognitive development, then bidirectional linkages will be found between all sensory modalities and frontal-parietal control hierarchies, so infusing all senses with somatic-motoric properties, thereby structuring all perception by relevant affordances, so solving frame problems for embodied agents. Drawing upon the Free Energy Principle and Active Inference framework, I describe a particular mechanism for intentional action selection via consciously imagined (and explicitly represented) goal realization, where contrasts between desired and present states influence ongoing policy selection via predictive coding mechanisms and backward-chained imaginings (as self-realizing predictions). This embodied developmental legacy suggests a mechanism by which imaginings can be intentionally shaped by (internalized) partially-expressed motor acts, so providing means of agentic control for attention, working memory, imagination, and behavior. I further describe the nature(s) of mental causation and self-control, and also provide an account of readiness potentials in Libet paradigms wherein conscious intentions shape causal streams leading to enaction. Finally, I provide neurophenomenological handlings of prototypical qualia including pleasure, pain, and desire in terms of self-annihilating free energy gradients via quasi-synesthetic interoceptive active inference. In brief, this manuscript is intended to illustrate how radically embodied minds may create foundations for intelligence (as capacity for learning and inference), consciousness (as somatically-grounded self-world modeling), and will (as deployment of predictive models for enacting valued goals).

## 1. Introduction

### 1.1. Descartes’ Errors and Insights

*“Any time a theory builder proposes to call any event, state, structure, etc., in a system (say the brain of an organism) a signal or message or command or otherwise endows it with content, he takes out a loan of intelligence. He implicitly posits along with his signals, messages, or commands, something that can serve a signal reader, message-understander, or commander, else his ‘signals’ will be for naught, will decay unreceived, uncomprehended. This loan must be repaid eventually finding and analyzing away these readers or comprehenders; for, failing this, the theory will have among its elements unanalyzed man-analogues endowed with enough intelligence to read the signals, etc., and thus the theory will postpone answering the major question: what makes for intelligence?”*

—Daniel Dennett [1]

From the traditional perspective of cognitive science, minds are understood as analyzable on multiple levels [2], where functional (or computational) properties can be considered separately from their specific algorithmic realizations, which can further be considered separately from particular implementational details. This multilevel approach allows progress to be made on studying mental functions without requiring understanding of underlying neurobiological processes, so allowing cognitive science to proceed without being held back by our limited understanding of nervous systems. Alternatively, combining different levels of analysis can provide constraints over plausible hypotheses, so affording inferential synergy.

Another perspective is provided by “4-E” cognition [3,4,5], in which minds are conceptualized as inherently embodied, embedded, extended, and enactive. From this point of view, understanding cognition requires considering how intelligent systems depend on bodily control processes. 4-E cognitive science further emphasizes how embedding within particular environments both enables and constrains functioning, where functional properties of mind extend into a world/niche that is modified/constructed via value-driven actions. More radical versions of this embodied-enactivist perspective tend to reject computational framings from traditional cognitive science, eschewing explicit models and representations in favor of dynamic environmental couplings. More traditional “cognitivists”, in contrast, tend to dismiss embodied cognition as a research program whose promise is limited by rejecting computational principles connecting brains and minds. From this point of view, embodied cognitive science is sometimes dismissed as a collection of interesting mind-body correlations, but which may be conceptually shallow in lacking precise operationalization.

While these perspectives often seem irreconcilable, there is near-universal agreement that cognitive science needs to divorce itself from the last vestiges of Cartesian thinking [6,7,8,9,10,11,12]. The only point of disagreement seems to be which aspects of Cartesian thinking are most egregiously mistaken. The charges are as follows:

1. The mind-body problem: Separating bodies and minds as distinct orders of being.
2. The theater fallacy: Describing perception in terms of the re-presentation of sensations to inner experiencers.
3. The homunculus fallacy: Failing to realize the inadequacy of inner experiencers as explanations, since these would require further experiencers to explain their experiences, resulting in infinite regress.

Many argue that the primary goal of cognitive science should be explaining away this naïve folk psychology in terms of non-mental computational and mechanistic processes [13,14]. Enactivists further (and differently) argue that cognitive science will only be thoroughly cleansed of its Cartesian origins once we eliminate concepts such as representation from our explanatory frameworks [3]. Yet the overwhelming consensus is clear: the mind sciences must rid themselves of the legacy of Descartes’ errors. The ghost must be exorcised from the machine.

Below I suggest this consensus may be mistaken along important dimensions, and propose ways in which each of these supposed errors point to invaluable perspectives. In brief:

1. Minds are thoroughly embodied, embedded, enacted, and extended, but there are functionally important aspects of mind (e.g., integrative processes supporting consciousness) that do not extend into bodies, nor even throughout the entire brain.
2. The brain not only infers mental spaces, but it populates these spaces with representations of sensations and actions, so providing bases for causal reasoning and planning via mental simulations.
3. Not only are experiences re-presented to inner experiencers, but these experiencers take the form of embodied person-models with degrees of agency, and even more, these quasi-homunculi form necessary scaffolding for nearly all aspects of mind.

In what follows, I intend to justify these claims and show how attention, imagination, and goal-oriented behavior may be explained using a Bayesian computational framework for understanding action, perception, and consciousness. My ultimate goal is illustrating how understanding the nature(s) of embodiment may allow for bridges between computational and enactivist perspectives on minds, so affording a grounding for unification in cognitive science.

### 1.2. Radically Embodied Minds

*“Now what are space and time? Are they actual entities? Are they only determinations or also relations of things, but still such as would belong to them even if they were not intuited? Or are they such that they belong only to the form of intuition, and therefore to the subjective constitution of our mind, without which these predicates could not be ascribed to any things at all?... Concepts without intuitions are empty, intuitions without concepts are blind… By synthesis, in its most general sense, I understand the act of putting different representations together, and of grasping what is manifold in them in one knowledge… The mind could never think its identity in the manifoldness of its representations… if it did not have before its eyes the identity of its act, whereby it subordinates all… to a transcendental unity… This thoroughgoing synthetic unity of perceptions is the form of experience; it is nothing less than the synthetic unity of appearances in accordance with concepts.”*

—Immanuel Kant [15]

*“We shall never get beyond the representation, i.e. the phenomenon. We shall therefore remain at the outside of things; we shall never be able to penetrate into their inner nature, and investigate what they are in themselves... So far I agree with Kant. But now, as the counterpoise to this truth, I have stressed that other truth that we are not merely the knowing subject, but that we ourselves are also among those realities or entities we require to know, that we ourselves are the thing-in-itself. Consequently, a way from within stands open to us as to that real inner nature of things to which we cannot penetrate from without. It is, so to speak, a subterranean passage, a secret alliance, which, as if by treachery, places us all at once in the fortress that could not be taken by attack from without.”*

—Arthur Schopenhauer [16]

Natural selection may have necessarily relied on general-purpose learning mechanisms for designing organisms capable of adaptively navigating (and constructing) their environments [17]. With respect to the importance of domain-general processes, Mountcastle [18] suggested a common algorithm for hierarchical pattern abstraction upon discovering the canonical layered-columnar organization of all neocortical tissue. Empirical evidence increasingly supports this suggestion, with hierarchical “predictive coding” (or predictive processing more generally) providing a unifying account of cortical functioning [19,20]. This dependence upon broadly applicable mechanisms may have been a matter of necessity due to the limits of genetic specification. While complex structures can be ‘encoded’ by genomes, particular phenotypes are realized in an algorithmic fashion, similar to how simple equations can generate highly complex fractal patterns [21,22]. For example, kidneys are complex, but no single nephron is special a priori. Similarly, brains have complex microstructure and macrostructure, but with few exceptions [23,24], no single neuronal connection is special a priori; rather, most neural complexity arises through experience-dependent self-organization. Further, much of the functional significance of specific connections in complex neural networks may be inherently difficult to predict due to the sensitivity of (chaotic) self-organizing systems to initial conditions [25,26]. Predicting functional significances may be even more limited to the degree that ‘representational’ properties of networks are shaped by information that will only emerge through unique developmental experiences.

In these ways, while some predictable features of brains may be subject to extensive genetic canalization [27,28,29], evolution may have been unable to produce cognitive adaptations relying on pre-specified complex representations. Yet, empirically, infants seem to possess impressively rich knowledge of objects and processes [30,31]—though developmental studies usually occur at several months post-birth, and even newborns have prenatal learning experiences [32]. Even largely empiricist statistical learning models from “Bayesian cognitive science” acknowledge the need for inborn inductive biases to facilitate inference and learning [33,34,35]. However, if there are substantial limits to genetic specification, how is this prior knowledge introduced?

I suggest the problems of under-constrained inference spaces are solved by remembering that brains evolved and develop as control systems for bodies, the regulation of which continues to be the primary task and central context of minds throughout life [36,37]. Bodies represent near-ideal initial systems for learning and inference, with this prototypical object and causal system providing bases for further modeling. Several factors contribute to the power of embodied learning [38,39]:

1. Constant availability for observation, even prenatally.
2. Multimodal sensory integration allowing for ambiguity reduction in one modality based on information within other modalities (i.e., cross-modal priors).
3. Within-body interactions (e.g., thumb sucking; hand–hand interaction; skeletal force transfer).
4. Action-driven perception (e.g., efference copies and corollary discharges as prior expectations; hypothesis testing via motion and interaction).
5. Affective salience (e.g., body states influencing value signals, so directing attentional and meta-plasticity factors).

Support for cross-modal synergy may be found in studies of adults learning motor sequences where performance is enhanced by combining multiple modalities [40,41,42]. Other insights regarding the nature of embodied learning derive from studies of developmental robotics and infant development [39,43], wherein morphological constraints and affordances function as implicit inductive biases for accelerated learning. For example, the limited range of motion of shoulder joints may increase tendencies for situating objects (beginning with hands themselves) in locations where they can be more readily explored with other sensor and effector systems [38].

By this account, complex minds necessarily require initial experiences of learning to control bodies, with increasing levels of complexity achieved—over the course of evolution and development—by expanding hierarchically-higher cortical areas [44]. This somatic developmental legacy is consistent with accounts in which abstract symbolic thought is grounded in mental simulation [45,46] and metaphorical extension from embodied experiences [47,48]. Below I will further characterize these embodied foundations for minds, suggesting that associative linkages to sensors and effectors generate body maps at multiple levels of abstraction, ranging from 1st-person semi-transparent interfaces [49] to 3rd-person body schemas capable of acting as self-reflexive intentional controllers (i.e., teleological agents).

### 1.3. The Cybernetic Bayesian Brain

*“Each movement we make by which we alter the appearance of objects should be thought of as an experiment designed to test whether we have understood correctly the invariant relations of the phenomena before us, that is, their existence in definite spatial relations.”*

—Hermann Ludwig Ferdinand von Helmholtz [50]

[Note: While the following section might be technically challenging, the key takeaway is that all cortex may operate according to a common algorithm of “*free energy” minimization* via *hierarchical predictive processing (HPP) (cf. predictive coding)*, in which prior expectations generate *top-down predictions* of likely observations, and where discrepancies between predictions and observations ascend to hierarchically higher levels as *prediction errors*. Biasing the degree to which prediction errors are likely to be passed upwards is referred to as *precision weighting*, which is understood as constituting attentional selection for Bayesian inference via hierarchical predictive processing.]

As perceptual illusions demonstrate [51,52], information arriving at the senses is inherently ambiguous, in that similar inputs could result from an unbounded number of world states (e.g., is an object small and close, or large and distant?). The *Bayesian brain hypothesis* states that perception can be understood as a kind of probabilistic inference, given sensory observations and prior expectations from past experience [53]. These inferences are hypothesized to be “Bayesian” in constituting a weighted combination of priors and likelihood mappings between observations and their hidden (or latent) causes from world states. Along these lines, the *Free Energy Principle and Active Inference (FEP-AI)* framework offers a promising integrative perspective for describing both perception and action in terms of probabilistic inference and *prediction error minimization* [54,55,56]. FEP-AI suggests that hierarchically-organized nervous systems entail hierarchical generative models, wherein perception (as inference) is constituted by probabilistic estimates (or predictions) of the likely causes of sensory observations.

The FEP-AI framework is grounded in fundamental biophysical considerations [57,58], as well as principles of *cybernetics: the analysis of complex adaptive systems in terms of self-regulation/governance with varying forms of feedback* [59,60,61]. Persisting systems must regulate both internal and external states to avoid entropic accumulation, which the “Good regulator theorem” suggests requires some kind of (predictive) modeling in order to ensure adaptive selection [36]. Prediction error—also referred to as “*free energy*”, or “surprisal”—can be minimized either by updating the implicit model of system-internal dynamics (i.e., perceptual inference), or by modifying external dynamics to make sensory-input more closely match predictions (hence, active inference). In this way, perception and action are both means of maximizing model-evidence (by minimizing prediction error) for the implicit prediction of system-preserving states, a process referred to as “self-evidencing” [62]. Intriguingly (and perhaps strangely) [63,64], the general logic of this kind of analysis appears consistent with pre-theoretic philosophical intuitions in which persisting systems are viewed as possessing a kind of ‘will-to-exist’ [65,66], even if this apparent goal-directedness is actually illusory (i.e., teleonomy, rather than actual teleology) [13]. While deflationary accounts of teleological phenomena emphasize continuity with teleonomical processes [14], the purpose of this manuscript is to single out and explain not just the origins of goal-directedness, but to make inroads into understanding uniquely human-like intentionality.

**Figure 1:**
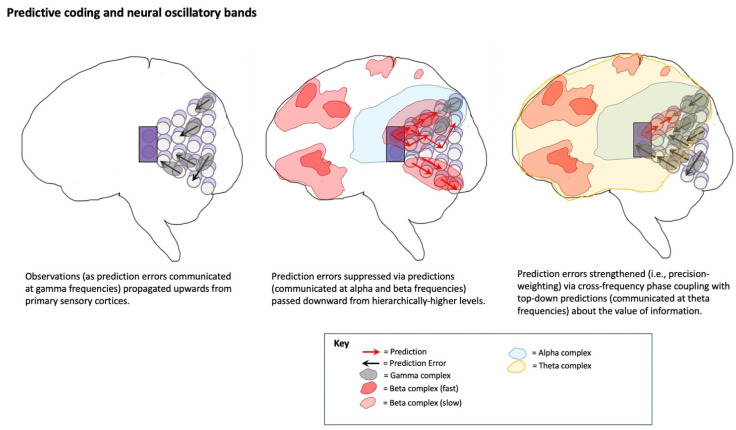
A schematic of hierarchical predictive processing in the brain. Left panel: Observations from primary sensory modalities (black arrows) indicate messages passed hierarchically upwards via superficial pyramidal neurons, communicated via small synchronous complexes (i.e., neuronal ensembles) at gamma frequencies. Middle panel: Predictions from hierarchically deeper areas of the brain (red arrows) suppress ascending observations, communicated via synchronous complexes of varying sizes at alpha and beta frequencies; bottom-up observations (as prediction errors) are only passed upwards when they fail to be anticipated by top-down predictions. Right panel: Attentional selection via strengthening of prediction errors by expectations regarding value of information, communicated via cross-frequency phase coupling with large synchronous complexes at theta frequencies. For all panels, darker arrows indicate degree of precision weighting associated with entailed (implicit) probabilistic beliefs, so determining relative contributions to Bayesian inference/updating. Please see previous work for more details on these hypothesized biocomputational principles [73,74].

HPP provides a parsimonious account of how this Bayesian world-modeling may be realized on algorithmic and implementational levels of analysis [19]. In HPP (Figure 1), top-down (empirical) priors are passed downwards as predictions based on posterior expectations (i.e., beliefs revised after making observations), which suppress bottom-up prediction errors from being transmitted up cortical hierarchies. In this encoding scheme, all observations take the form of prediction errors, indicating sensory inputs at the lowest hierarchical levels, sensory expectations at somewhat higher levels, and beliefs of a more folk psychological variety at even higher levels. [In these models, posterior expectations—or more generally beliefs—are formally equivalent to empirical priors at intermediate levels in the model; I will use (empirical) priors and posteriors interchangeably.] By only passing prediction errors up cortical hierarchies, predictive coding automatically prioritizes novel ‘news-worthy’ information in the process of updating beliefs and subsequent predictions. This recurrent message-passing is suggested to occur simultaneously in every part of cortex, with hierarchical dynamics reflecting hierarchical world structure [67,68], including events unfolding over multiple (hierarchically-nested) temporal scales [69,70,71]. In this way, HPP generates a dynamic mapping between brain and world, mediated by (hierarchically-organized) cycles of action-influenced perception. HPP further provides a mechanistic process model for enaction in FEP-AI by providing means of altering world states to better fit predictions via active inference [72]. This means that all neuronal dynamics and ensuing action can be regarded as complying with the same imperative: namely, to minimize prediction error (i.e., free energy, or “surprisal”).

According to HPP, brains function as both cybernetic controllers and memory systems [59,60,61], with experience-dependent expectations providing bases for control, which in turn create new memories and predictions. This cybernetic perspective has been further extended to interoceptive inference [61,75] in terms of homeostatic maintenance via predictive regulation (i.e., allostasis). In this account of emotional experience, affective states arise from active inferential control of interoceptive and autonomic states under different levels of uncertainty [75,76].

Reliable inference must account for degrees of certainty associated with various beliefs, which in HPP is described as “precision” (i.e., inverse variance) of probability distributions [77]. In HPP, ascending signals update descending posterior expectations proportional to relative precisions of (empirical) prior predictions and sensory-grounded observations. More precise prediction errors have greater influences in updating higher-level beliefs, which can be thought of as selecting more reliable sources of ‘news’–as opposed to more unreliable, or ‘fake’ news. Algorithmically, certainty-based biasing of prediction errors realizes Bayesian inference as a precision-weighted sum of probabilities, so providing a functional basis for attentional selection. Mechanistically (and potentially phenomenologically), this attentional selection involves modulation of excitation for particular neuronal populations, so making entailed precision-weighted prediction errors more or less likely to percolate into deeper portions of cortical hierarchies where this information may shape larger-scale (potentially conscious) dynamics [73,74].

Precision weighting can have profound effects on relative influences of descending predictions and ascending prediction errors. If bottom-up signals are given too much precision, then excessive sensory prediction errors may access deeper portions of cortical hierarchies, which could potentially result in the kinds of overly intense sensory reactions often observed with autism [78,79,80]. Alternatively, if bottom-up signals are given too little precision, then prediction errors may not result in belief updating, which if excessive, could result in false-positive inferences, potentially including the kinds of delusions and hallucinations observed with schizophrenia [81,82,83].

Between the basic idea of perception as inference and its cybernetic extensions to active inference, the Bayesian brain is thoroughly embodied. This discussion goes further in suggesting that action-oriented body maps form the core of Bayesian brains, structuring inferential flows in ways that not only enhance control, but also allow minds to solve inferential problems that have hitherto been assumed to require extensive innate knowledge. As described above, bodies provide brains with learning opportunities in which hypothesis spaces are fruitfully constrained, and so rendered tractable. In light of the adaptive significance of embodied learning, selective pressures are likely to shape bodies in ways that brains readily infer and learn, so shaping further histories of selection. I further suggest this more easily acquirable knowledge allows learners to handle increasingly challenging problems (or lessons [84]) along zones of proximal development [85].

Neurodevelopmentally, this model can be considered broadly Piagetian [86], albeit without intellectual commitments with respect to particular developmental stages. This point of view is consistent with perspectives in which body-centric self-models are required for successful structure learning in the process of developing reasonably accurate and useful predictive models [75,87,88]. This proposal is also consistent with previous descriptions of active inference [89], but suggesting a particular—and I suggest, necessary—means by which generative models come to reflect world structure. That is, we may acquire many foundational (empirical) priors from learning about bodies as more tractable causal (and controllable) systems. Without this toehold/grip with respect to inferential bootstrapping, it may be the case that neither Bayesian cognitive science nor Bayesian brains could explain how biological learners handle under-constrained inference spaces.

The notion of embodiment as a source of foundational beliefs is increasingly recognized in FEP-AI. Allen and Tsakiris [90] have compellingly proposed a “body as first prior” model in which interoceptive inference provides a source of highly precise priors (or predictions), so allowing overall active inferential belief dynamics to be dominated by organismic, allostatic needs. In their account, interoception supplies fundamental priors in yet another sense in playing central roles with respect to establishing models of body ownership and (minimal) selfhood, both of which constitute necessary preconditions for learning about other aspects of the world. The specific nature(s) of these embodied priors has been further explored in terms of their shaping by developmentally early socioemotional coupling, including with respect to perinatal and prenatal interactions with caregivers upon which infants depend for life [87,91,92]. Below, I explore some of these ideas, as well as additional (complementary) ways in which embodiment may form necessary foundations in growing minds, the extent of which may be difficult to overstate.

## 2. From Action to Attention and Back Again

While some of the content in these next sections may be challenging, the key messages from these sections are as follows:

1. Much of conscious goal-oriented behavior may largely be realized via iterative comparisons between sensed and imagined states, with predictive processing mechanisms automatically generating sensibly prioritized sub-goals based on prediction errors from these contrasting operations.
2. Partially-expressed motor predictions—initially overtly expressed, and later internalized—may provide a basis for all intentionally-directed attention, working memory, and imagination.
3. These imaginings may provide a basis for conscious control of overt patterns of enaction, including the pursuit of complex goals.

### 2.1. Actions from Imaginings

The goal of this manuscript is to illustrate the radically embodied foundations of agency, ranging from basic motor control to complex planning. Towards this end, I propose a model in which all conscious goal-directed behavior is realized with hierarchical predictive coding and iterated comparisons among perceptions of sensed and imagined (i.e., counterfactual) states [93]. Let us consider someone writing a manuscript at a computer and discovering that they want tea, while also inferring that their cup is empty. These experiences would likely include mental imagery or memories of drinking tea, accompanied by feelings of thirst. However, such counterfactual beliefs (or predictions) would then be contradicted by sensory evidence if tea is not presently being consumed. The contrast between the counterfactual tea drinking and the observation of an empty cup would then be likely to prime similar situations in the past (e.g., unresolved thirst or hunger). Those situations will also be likely to be accompanied by relevant affordances [94,95,96] (e.g., tea-making/acquiring actions) associated with minimizing those varieties of discrepancies between preferred and present-estimated states. That is, memories and analogous imaginings are likely be dominated by actions whose relevance is determined based on past similar situations [59,97].

**Figure 2:**
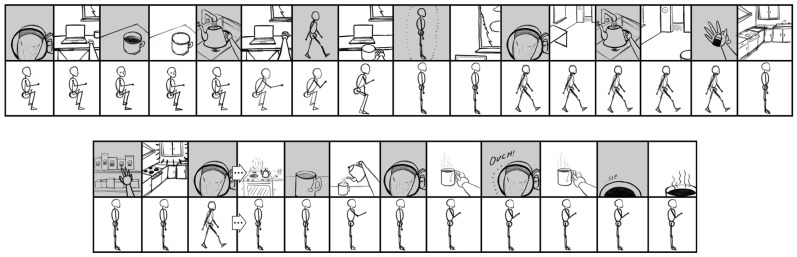
Imaginings and perceptions associated with policy selection via backward chaining from goal states. Top panels of each row illustrate the counterfactual predictions (grey) and observations (white) listed in Table 1. Bottom panels of each row depict associated body positions. Note: This example lacks substantial metacognitive and reflexive processing, with only a few panels depicting the agent imagining itself from an external viewpoint. To the extent that consciousness actually models the actions associated with making tea (as opposed to mind-wandering), a more immediate and non-reflective mode of cognition might be expected for this kind of relatively simple behavior. However, for more complex goals, we might expect more elaborate imaginings involving objectified self-representations with varying levels of detail and abstraction.

These counterfactual imaginings will be likely to be centered on goal-specific discrepancies, such as the fact that one may be sitting in front of a computer, rather than acquiring the desired tea (Table 1; Figure 2). In this case, the most likely set of affordances to be retrieved from memory would involve actions such as ambulating to the kitchen, where the sink, stove, and tea kettle are located. However, our thirsty agent may find themselves confronted with yet another set of discrepancies, such as the fact that sitting is not walking to the kitchen. In this case, the next likely set of memory-affordances to be retrieved could be those involving getting up, and perhaps shifting weight and pressing one’s feet into the ground. At various points, these counterfactual plans may become sufficiently close to the present state that they become actionable, and so contribute to ongoing action selection.

Mechanistically speaking, this actionability of counterfactual imaginings may be realized when neuronal ensembles associated with goal representations have relatively high degrees of overlap with those associated with proximate sensorimotor contingencies. If critical thresholds for motoric action selection are surpassed under such conditions of convergent excitation between present-estimated and desired states, then neural activity from imagined goals may become capable of functionally coupling with—or directionally entraining (i.e., “enslaving”) [98]—an organism’s effector systems. These imagined scenarios will also be continuously altered based on changing sensory evidence with unfolding behavior. For example, the location of the tea kettle may come into view en route to the kitchen, along with memories related to filling and emptying the kettle, so adjusting expectations with respect to whether the kettle needs to be brought to the sink to obtain water.

In FEP-AI [55], the sequences of actions (i.e., policies) we infer ourselves enacting are dominated by our prior preferences and expected consequences of actions. Crucially for adaptive behavior, this imperative to minimize prediction error (i.e., free energy) can also be applied to expected prediction error (i.e., expected free energy), wherein we select policies (potentially implicitly) anticipated to bring about preferred outcomes (e.g., having a cup of tea) in the future. This expected free energy (i.e., cumulative, precision-weighted prediction error) can be further decomposed based on relevance to either pragmatic or epistemic value, where pragmatic affordance is defined in terms of prior preferences (i.e., drinking tea) and epistemic affordance entails opportunities for reducing uncertainty (e.g., locating teabags) [99].

To the extent that actions are highly rehearsed, minimal conscious visualization may be required for goal attainment [43,100]. If tea is central to the lifeworld of our agent [101,102], then the entire sequence could end up proceeding with only very brief flashes of subjective awareness [103]. It is also notable that little awareness will likely accompany the coordinated activity of specific muscles, for which effortless mastery will be attained early in development. To the extent that goal-attainment involves novel circumstances—e.g., learning how to prepare loose-leaf tea for the first time—consciousness may play more of a central role in shaping behavior.

In this model of imaginative planning, activation of goal-related representations produces prediction errors wherever there are discrepancies between anticipated goal states and inferred present states. That is, goal-related representations act as predictions, and discrepancies with estimated present states result in prediction errors within particular sub-representations related to goal-attainment, generated at multiple hierarchical levels. When goal-discrepancy prediction errors are passed up the cortical hierarchy, they may access more richly connected networks, allowing for (potentially conscious) global availability of information [104], and so become more effective at driving subsequent neuronal activity. Given sufficient experience, goal-related representations with greater activity at the next moment will likely correspond to neuronal ensembles that most reliably succeeded (and so were reinforced) in minimizing those particular kinds of discrepancies in the past (i.e., relevant affordances).

By this account, comparisons between representations of goal states and present states generate greater activity for goal-related representations with more prediction error, often corresponding to the largest obstacles to goal attainment. These sources of maximal prediction error from iterative contrasting may naturally suggest prioritization for selecting appropriate sub-goals for overall goal-realization [105]. Sequential comparisons between representations of sub-goals and estimated present states will likely activate relevant sub-representations for additional obstacles, the overcoming of which becomes the next goal state. This comparison process proceeds iteratively, with repeated discrete updating [106] of imagined goals and estimated present states, so shaping neural dynamics (and entailed streams of experience) in accordance with predicted value realization.

With experience and learning—including via imagined experiences [107]—this iterative selection process is likely to become increasingly efficient. Considering that superordinate and subordinate action sequences are themselves associatively linked, they will provide mutual constraints as parallel comparisons continuously minimize overall prediction errors on multiple levels of action hierarchies. Thus, similar cognitive processes may be involved in selecting higher-level strategies for (potentially abstract) goal attainment, as well as the conscious adjustment of lower-level sequences retrieved from memory for intentional motor control. In terms of active inference, skillful motoric engagement is largely achieved through the ability of predicted actions to provide a source of “equilibrium points” [108], realized as neural systems dynamically self-organize via predictive processing mechanisms [109]. The model presented here describes a particular (potentially conscious) source of such high-level predictions as drivers of behavior. [Notably, the existence of separate dopamine value signals in the ventral tegmental area and substantia nigra pars compacta [110]—along with differing temporal dynamics and credit assignment challenges—suggest complexities requiring additional neurocomputational details in order to adequately describe (hierarchical) neuronal activity selection.] The imagination-focused account above describes the operation of intentional control processes to the (limited) degree they are capable of influencing behavior. Often this intentional influence may ‘merely’ take the role of biasing competition and cooperation among unconscious habitual and reflexive patterns.

By this account, to have a goal is to predict its realization, where initial predictions generate further causal paths as means of bridging gaps between imagination and reality. This kind of connection between imagination and action has precedents in ideomotor theory [111,112,113], which has also been explored in active inferential terms with respect to attentional biasing (i.e., precision weighting) [114]. Below I expand on this work in proposing that all voluntary (and much involuntary) attention may be realized by partially-expressed motor predictions as mental actions, so providing an agentic source for precision weighting in governing inferential dynamics as a kind of covert motoric skill. [Please note that I do not intend to suggest that most attention is consciously directed. Rather, much (and perhaps most) top-down precision weighting might be automatically generated by interoceptive salience maps, implemented by insular and cingulate cortical hierarchies [115].

### 2.2. Attention from Actions

*“A good way to begin to consider the overall behavior of the cerebral cortex is to imagine that the front of the brain is ‘looking at’ the sensory systems, most of which are at the back of the brain. This division of labor does not lead to an infinite regress… The hypothesis of the homunculus is very much out of fashion these days, but this is, after all, how everyone thinks of themselves. It would be surprising if this overwhelming illusion did not reflect in some way the general organization of the brain.”*

—Francis Crick and Christoff Koch [6]

**Figure 3:**
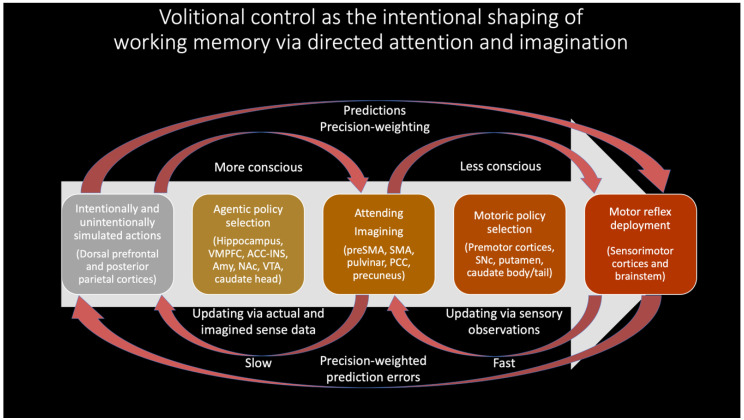
Imaginative policy selection via a multilevel active inferential control hierarchy and associated neural systems. Going from left to right, predictions are passed downwards as (empirical) prior expectations, and are updated into posterior expectations (and subsequent priors) by sensory observations, which are then passed upwards as prediction errors. Upper-level control processes (left action-perception cycle) involve more slowly-evolving attracting states, corresponding to more coarse-grained, higher-level abstract modeling of organismic-scale causes, which may be associated with conscious intentionality. Lower-level control processes (right action-perception cycle) involve more quickly-evolving attracting states, allowing for rapid adjustment of action-perception cycles and fine-grained environmental coupling. While multiple factors may point to the significance of a two-tier hierarchy, this distinction ought not be overstated, as integrating (potentially conscious) processes may potentially attend to (or couple with) dynamics from either level. VMPFC = ventromedial prefrontal cortex, ACC-INS = anterior cingulate cortex and insula, Amy = amygdala, NAc = nucleus accumbens, VTA = ventral tegmental area, SMA = supplementary motor area, PCC = posterior cingulate cortex, SNc = substantia nigra pars compacta.

In this radically embodied account of attentional control, partially expressed motor predictions realize all intentional directing of perception, including with respect to attention, working memory, imagination, and action. This control is achieved by efferent copies from action-related neuronal ensembles to associated perception-related neural populations, with functional linkages established via past learning [116,117]. Developmentally—and evolutionarily [118]—actions initially take the form of externally expressed behavior; with respect to overt attention, effector systems orient sensors relative to the environment and so change patterns of sensation. However, via either incomplete or inhibited expression, these actions will also be expressed covertly in imagination as mental simulations with varying degrees of detail and awareness. When these partially-expressed motor predictions for overt attending are activated, connections to associated perceptual components can then be used as bases for covert attending. With experience, adaptive control over overt and covert expression will be learned, so allowing context-sensitive shifting between perception, imagination, and action. Further degrees of control over perception and action can be enabled by intentionally directing attention to contents of working memory (Figure 3), including with respect to the imagination of counterfactual scenarios required for causal reasoning and planning [119].

This model represents a generalization of Vygotsky’s [120] hypothesis regarding the development of thinking through the internalization of speech. By this account, first we learn how to speak, then we learn how to prepare to speak without overt expression, and then by learning how to internally speak to ourselves—imagining what we would have heard if speech were externally expressed—we acquire capacities for symbolic thought. Similarly, through the internalization of initially overt actions [121], all voluntary (and much involuntary) cognition may develop as a control hierarchy grounded in controllable effector systems. Indeed, I propose skeletal muscle is the sole foundation for all voluntary control due to its unique ability to generate gross actions with real-time low-latency feedback.

To summarize, ontogenetically (and phylogenetically), information acquisition is initially biased via overt action-perception. However, learners eventually acquire the ability to perform actions covertly, and thereby utilize the associated perceptual components of particular simulated actions as bases for covert processing (including counterfactual imaginings). In all cases, actions have their origins in control hierarchies over sensorimotor cortices—and associated striatal loops—whose dynamics are grounded in manipulating skeletal muscles, along with associated sensations. In this way, partially-expressed motor predictions can bias attention and working memory spatially (e.g., simulated saccades), temporally (e.g., simulated rhythmic actions), or even based on semantic or object feature information (e.g., simulated speech) (Table 2).

### 2.3. Imaginings from Attention

This account is consistent with premotor [122] and biased competition [123] theories of attention. However, I further suggest partially-expressed motor predictions are the only means by which content is voluntarily generated in working memory (Figure 3), whether based on attending to perceptual traces of recent sensations, or generating counterfactual perceptual experiences decoupled from actual sensory stimulation (i.e., imagination). While this proposal may seem excessively radical in the extent to which embodiment is emphasized, convergent support can be found in substantial evidence implicating the “formation of internal motor traces” in working memory [124]. Further evidence may be obtained in attentional selection being enhanced when neuronal oscillations from frontal eye fields entrain sensory cortices [125], as well as from visual attention and working memory heavily depending on frontal-parietal networks [126,127] (which are here interpreted as upper levels of action-perception hierarchies). With respect to embodied sources of top-down attention, striatum and midbrain value signals (e.g., dopamine) likely play key roles [128], both influencing moment-to-moment pattern selection, and also allowing future planning to be influenced by histories of reinforcement and punishment. To the extent that learning effectively aligns these patterns with agentic goals, mental content—and the resultant influences on action selection—can be understood as involving intentionality.

**Figure 4:**
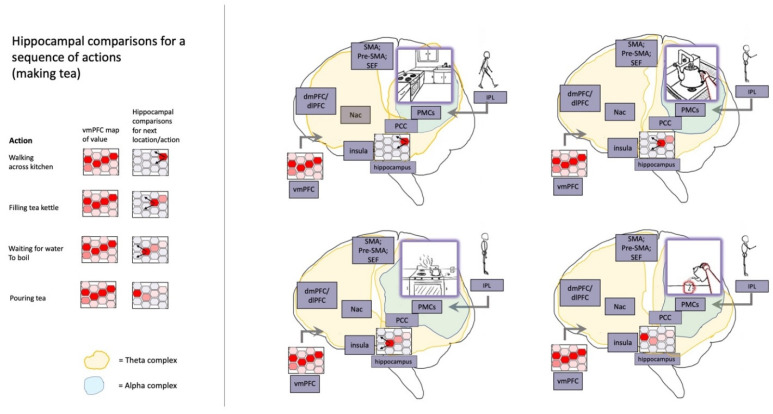
*Reprinted with permission from Safron*, 2020b. Hippocampally-orchestrated imaginative planning and action selection via generalized navigation. Action sequences from Figure 2 are depicted with respect to relevant neural processes. The hippocampal system provides (a) organization of cortical attracting states into value-canalized spatiotemporal trajectories, (b) stabilization of ensembles via theta-mediated cross-frequency phase coupling, and (c) goal-oriented cognition and behavior via contrasting (not depicted) sensed and imagined states. Hippocampal trajectories are shaped according to whichever paths are expected to result in more positively valanced outcomes (cf. reward prediction errors). The expected value associated with navigating to different portions of (potentially abstract) space is informed via coupling with similarly spatiotemporally-organized value representations (red shaded hexagons) in vmPFC and associated systems. As chained patterns of activity progress across hippocampal place fields (red hexagons with variable degrees of shading), theta-synchronized frontal ensembles (yellow shading spreading towards the front of the brain) help to generate (via cross-frequency phase coupling) ensembles for directing attention, working memory, and overt enaction. Sensory updating of posterior cortices occurs at alpha frequencies (blue shading), so providing a basis for conscious perception and imagination. With respect to these integrated estimates of sensory states, hippocampal coupling at theta frequencies (yellow shading spreading towards the back of the brain) provides a basis for (a) episodic memory and replay, (b) novel imaginings, and (c) adjustment of neuronal activity selection via orchestrated contrasting between cortical ensembles. Abbreviations: nAC = nucleus accumbens; vmPFC = ventromedial prefrontal cortex; dmPFC = dorsomedial prefrontal cortex; SMA = supplementary motor area; Pre-SMA = presupplementary motor area; SEF = supplementary eye fields; PCC = posterior cingulate cortex; PMCs = posterior medial cortices; IPL = inferior parietal lobule.

Imagined goals may be generated and contrasted with estimated states (whether imagined or observed) on timescales of approximately 200–300 msec [129,130,131,132], potentially implemented by activation/stabilization of neocortical ensembles via cross-frequency phase coupling with hippocampal theta rhythms (Figure 4) [133,134]. The iterative generation of new (posterior) goal-relevant imaginings—may take significantly longer, potentially depending in complex ways in which processes are contrasted. If this process requires stabilization of novel combinations of cortical ensembles by the hippocampal complex, then this may help to explain why medial temporal lobe damage is associated with impaired counterfactual processing [135,136], which here forms the basis of intentional action selection via iterative contrasting and predictive processing. A prediction of these models is that hippocampal damage may be associated with disrupted goal-pursuit in dementia—above and beyond the problem of task-forgetting—for which additional anecdotal evidence can be found with the case of the neurological patient “HM” [137]. The central role of the hippocampus for orchestrating goal-oriented behavior is further suggested by its involvement in “vicarious trial-and-error” behavior [138], as well as by the centrality of theta rhythms for intentional control [126,131,139,140]. Additional supporting evidence can be found in hippocampally-mediated orchestration of counterfactual inferences in other domains, ranging from predictive information over likely trajectories for locomoting rodents [141,142] to the simulation of alternative perspectives by imagining humans [143,144].

These proposals expand on previous descriptions of motor control via predictive processing [114] by emphasizing the role of consciously-experienced body maps as a source of intentionally-directed attention (i.e., precision weighting), imagination, and action. However, if voluntary action is a function of attention, and if attention is achieved by simulated actions and partially-expressed motor predictions, then what allows voluntary actions to develop in the first place? This potential explanatory regress is prevented by the (potentially surprising) ease of controlling cleverly ‘designed’ body plans, particularly when such morphologies are constrained to adaptive areas of state space [38,110]. For example, much of locomotion emerges from relatively controllable pendulum dynamics, and brainstem and spinal pattern generators further help produce coherently timed force vectors and locomotory modes [145]. To provide another example, limited range of motion for shoulder, arm, and finger joints promote effective engagement and exploration of the world via grasping (e.g., gripping made easier by fingers not bending backwards) and manipulation within likely fields of view (e.g., arms being more likely to place objects in front of facial sensors). Such near-optimal grips may be further facilitated by the functional resemblance between finger pads and deformable soft robotics manipulators, where degrees of force provide adaptively adjustable contact surfaces, so simplifying control via offloading to morphological ‘computation’ [146]. By this account, not only do well-designed body plans automatically contribute to adaptive behavior [147], but such embodied intelligence provides foundations and scaffolding for all cognitive (and affective) development. These favorable learning conditions are further enhanced via supervision by other more experienced humans (including nurturing parents) in the context of human-engineered environments [92,148,149]. In these ways, we automatically find ourselves in capable bodies in the midst of value-laden goal-oriented activities [100], where these grips on the world eventually allow us to construct coherent world models and conscious intentionality.

## 3. Grounding Intentionality in Virtual Intrabody Interactions and Self-Annihilating Free Energy Gradients

*“We have to reject the age-old assumptions that put the body in the world and the seer in the body, or, conversely, the world and the body in the seer as in a box. Where are we to put the limit between the body and the world, since the world is flesh? Where in the body are we to put the seer, since evidently there is in the body only "shadows stuffed with organs," that is, more of the visible? The world seen is not "in" my body, and my body is not "in" the visible world ultimately: as flesh applied to a flesh, the world neither surrounds it nor is surrounded by it. A participation in and kinship with the visible, the vision neither envelops it nor is enveloped by it definitively. The superficial pellicle of the visible is only for my vision and for my body. But the depth beneath this surface contains my body and hence contains my vision. My body as a visible thing is contained within the full spectacle. But my seeing body subtends this visible body, and all the visibles with it. There is reciprocal insertion and intertwining of one in the other...”*.

—Maurice Merleau-Ponty [150]

This proposal is radically embodied in claiming to provide an exhaustive account of intentional control via internalized action patterns. Partially-expressed motor predictions are suggested to be the only means of volitional control over attention, working memory, and imagination, whether such influences are based on attending to a perceptual trace of recent sensations, or through generating novel counterfactual perceptual experiences via associated fictive actions. Representations selected by these partially-expressed motor predictions function as particularly robust predictions in active inference—perhaps particularly if made conscious [73]—so providing powerful means of voluntarily shaping thought and behavior.

In this active inferential view, *intentions* represent a functional intersection of beliefs and desires, where *desires* are understood as a species of *counterfactual beliefs*, so generating prediction errors (or *free energy gradients*) to be minimized through enaction. As will be discussed in greater detail below, emotions and feelings may be fruitfully conceptualized as the active and perceptual components of action-perception cycles over organismic modes. In this view, desires may be conceptualized as both *emotions as driving active inference* and also *feelings as updating perceptual models* [151]. As described above, the imagination of counterfactual desired world states will produce goal-relevant prediction errors, which are minimized either via updating predictions (desire as feeling), or by updating world states (desire as emotion).

**Figure 5:**
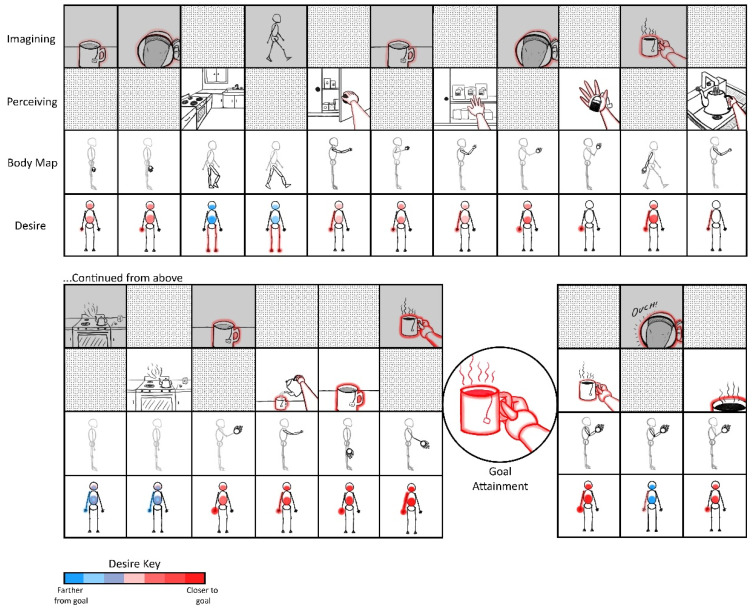
Interacting modalities in the context of imaginative planning and policy selection. This sequence of frames depicts interactions between modalities as agents select actions in order to achieve the goal of having tea (see Figure 2 and Figure 4). Each row depicts a different aspect of experience, all of which interact in the context of goal-oriented cognition and behavior. Imagining and Perceiving (1st and 2nd rows) correspond to the current content of visuospatial awareness, likely mediated by hierarchies centered on posterior medial cortices. Whether this workspace is occupied by perceiving or imagining would respectively be a function of either stronger interactions with hierarchically lower cortical areas, or more stimulus-decoupled default mode processing (so affording counterfactual percepts). Body map (3rd row) corresponds to experienced proprioceptive pose, likely mediated by a hierarchy centered on inferolateral parietal cortices. Differential shading and size of body parts indicate differential attentional focus and modeling properties associated with affordance-related salience with respect to ongoing goal pursuit. Desire (4th row) corresponds to affective body experiences, likely also mediated by inferolateral parietal networks, but also involving interactions with insula and cingulate cortices. Differential red and blue shading respectively indicate positive and negative valence associated with different body parts, including with respect to interoceptive estimates of semi-localized aspects of the internal milieu. Taken together, rows 1 and 2 could be considered as constituting the “mind’s eye” (or “Cartesian theater”), and rows 3 and 4 as the “lived body.” Through their coupling, these networks and associated phenomena may (potentially exhaustively) constitute physical substrates of consciousness as integrative workspace for agent-based modeling and control.

Given that sources of value associated with desires are rooted in homeostatic imperatives, these affectively-laden prediction errors will center on interoceptive modalities [152,153] (Figure 5). As compellingly described by Seth et al. [154] with respect to the insular inferential hierarchy, this predominantly interoceptive free energy may be allostatically minimized via modulating neuroendocrine and autonomic functions. Alternatively, these primarily interoceptive free energy gradients (here understood as desires) could be minimized through the more indirect strategy of generating counterfactual predictions regarding the exteroceptive and proprioceptive consequences of action [75]. If counterfactual proprioceptive poses are stably held in mind, they may eventually result in the driving of motor pools as neural systems self-organize to minimize prediction error via overt enaction [72,109,155]. From this perspective, all actions are ultimately understood as a kind of extended allostasis in constituting predictive homeostatic life-management [156].

The degree to which desires drive overt action selection via proprioceptive predictions will largely depend on differential precision weighting allocated to various portions of cortical hierarchies (Figure 1 and Figure 3). With respect to the insula, precision weighting could allow prediction errors to reach hierarchically higher (i.e., more anatomically anterior) levels [154], where interoceptive information may have more opportunities to influence predictions for exteroceptive and proprioceptive hierarchies, and thereby drive action. Whether overall prediction error (i.e., free energy) is minimized by updating internal models (i.e., perceptual inference) or updating world states (i.e., active inference) will depend on attenuating precision at primary modalities [72,157], so protecting goal-related predictions from disruption (or updating) by discrepancies with present sensory data. For example, decreased precision on lower levels of interoceptive hierarchies could promote interoceptive active inference via autonomic functions (i.e., desire as unconscious emotion), since reduced gain on interoceptive sensations will allow associated representations to be more updatable via predictive coding mechanisms. Increased precision on middle levels of the interoceptive hierarchy, in contrast, would promote interoceptive information reaching the anterior insula and attaining more global availability (i.e., desire as conscious feeling). If these consciously-felt interoceptive states generate robust predictions for other modalities, and if sensory evidence does not have excess precision, then free energy will flow up interoceptive and into exteroceptive and proprioceptive hierarchies, thereby driving action to minimize overall prediction error (i.e., free energy). In these ways, desire (as free energy gradient) may be viewed as a force [158] that flows across multimodal body maps, which may result in overt enaction if these cascading predictions are sufficiently robust to result in minimizing prediction error via spinal motor pools and associated muscular effectors. Computationally speaking, these information flows would be constituted by patterns of precision weighting, either selecting specific predictions for enaction (e.g., relevant affordances for minimizing particular kinds of interoceptive prediction errors), or as hyperpriors influencing policy selection thresholds (e.g., modulating neuromodulatory systems).

This account of driving large-scale neuronal activity selection by visceral desires is consistent with interoceptive inferences being uniquely capable of enslaving cortex due to the highly stable (and so precise) nature of those predictions [90], which may have further entraining power via the high centrality of these subnetworks. These models are supported by numerous studies in which insula-cingulate connectivity is shown to be central for motivated cognition and behavior [159,160,161,162]. Further indirect supporting evidence may be found in voluntary actions being more frequently initiated during exhalations, where associated neural dynamics (i.e., readiness potentials) exhibit modulation by respiratory cycles [163]. Perhaps the most compelling evidence for these models of viscerally-driven action may be found in work by Zhou et al. [164], wherein organismic saliency models constituted the highest level of hierarchical control among resting state networks.

Much interoceptively-influenced biasing of attention and action selection may be unconscious. However, when these viscerally-grounded [130] prediction errors reach levels of cortical hierarchies where we become aware of them, then we can further attend to these sensations using efference copies (as predictions, or Bayesian priors) from exteroceptive and proprioceptive modalities. For example, we can (either overtly or imaginatively) visually scan through maps of the body and its interior, so modeling interoceptive contents by means other than the sensory channels that directly transmit this information from the internal milieu. This intentional attending to interoceptive states could then allow us to modulate the degree to which consciousness and action is influenced by feelings of desire. Theoretically, this mechanism could also provide enactive models of mindfulness practices such as “body scanning” or meditation on the breath [165,166,167].

This account of emotional regulation from directed attention to interoceptive states can also apply to attention to exteroceptive and proprioceptive modalities. Partially-expressed motor predictions may bias activity in these body representations (e.g., simulated foveations on hands), so influencing which actions are likely to be selected next (e.g., hands grasping in particular ways). While subject to multiple interpretations, some evidence for this model may be found in precision-estimation being influenced via functional interactions between theta power from frontal midline structures and beta power from frontal-parietal networks [127], which here may be (speculatively) interpreted as respectively indicating fictitious foveations interacting with other aspects of action-oriented body maps (Table 2). To the extent that partially-expressed actions provide bases for top-down attention, we may intentionally influence attention by attending to action-oriented body maps, so driving further patterns of attending and intending. Is there really room for intentionality in this cascade of converging cross-modal predictions? The answer to this question will depend on how we define intention, which here represents any instance of conscious desires being able to influence neuronal activity selection. Human-like intentionally can further be said to arise when these processes are driven by goals involving narrative self-models and associated concepts, as will be described in greater detail below.

## 4. The Emergence of Conscious Teleological Agents

[Note: In what follows, the word consciousness is used in multiple senses, sometimes involving basic subjective experience, and other times involving conscious access with respect to the knowledge, manipulability, and reportability of experiences [168]. Unless otherwise specified, these discussions can be considered to refer to both senses of consciousness. For a more thorough discussion of the physical and computational substrates of phenomenal consciousness, please see Integrated World Modeling Theory [73,74].]

### 4.1. Generalized Dynamic Cores

*“What is the first and most fundamental thing a new-born infant has to do? If one subscribes to the free energy principle, the only thing it has to do is to resolve uncertainty about causes of its exteroceptive, proprioceptive and interoceptive sensations... It is at this point the importance of selfhood emerges – in the sense that the best explanation for the sensations of a sentient creature, immersed in an environment, must entail the distinction between self (creature) and non-self (environment). It follows that the first job of structure learning is to distinguish between the causes of sensations that can be attributed to self and those that cannot… The question posed here is whether a concept or experience of minimal selfhood rests upon selecting (i.e. learning) models that distinguish self from non-self or does it require models that accommodate a partition of agency into self, other, and everything else.”*

—Karl Friston [88]

*“[We] localize awareness of awareness and dream lucidity to the executive functions of the frontal cortex. We hypothesize that activation of this region is critical to self-consciousness — and repudiate any suggestion that ‘there is a little man seated in our frontal cortex’ or that ‘it all comes together’ there. We insist only that without frontal lobe activation the brain is not fully conscious. In summary, we could say, perhaps provocatively, that (self-) consciousness is like a theatre in that one watches something like a play, whenever the frontal lobe is activated. In waking, the ‘play’ includes the outside world. In lucid dreaming the ‘play’ is entirely internal. In both states, the ‘play’ is a model, hence virtual. But it is always physical and is always brain-based.”*

—Allan Hobson and Karl Friston [11]

The cybernetic Bayesian brain has also been extended to phenomenology, suggesting possible explanations for qualitative aspects of experience ranging from the sense of agency to synesthetic percepts. A felt sense of “presence” (or subjective realness) is suggested to correspond to the successful predictive suppression of informative interoceptive signals evoked by autonomic and motor actions, producing a sense of agency in association with self-generated action [154]. Histories of self-generated actions allow for the “mastery of sensorimotor contingencies” [169], with the extent and variety of evoked affordance-related predictive abilities (i.e., “counterfactual richness”) determining degrees of presence associated with various aspects of experience [61].

Speculatively, counterfactual richness could contribute to perceptual presence via micro-imaginings that may be barely accessible to conscious awareness. That is, perception may always involve associated affordance relations, but where such imaginings may not be consciously accessible due to their fleeting nature (e.g., a single integrative alpha complex over posterior modalities failing to be more broadly integrated into a coherent causal unfolding). Yet, such simulated affordances may nonetheless contribute to attentional selection of different aspects of percepts and their multimodal associations (e.g., likely interoceptive consequences), so generating a penumbra of possibility accompanied by a particular sense of meaningfulness. This model of *phenomenality without accessibility* may be crucially important for understanding multiple aspects of agency, in which consciously experienced *isolated qualia* may potentially have strong impacts on minds in providing surprisingly rich sources of “unconscious” processing.

Alternatively, part of the reason that counterfactual richness is associated with perceptual presence may be because these (non-actual) affordance-related predictions fail to suppress bottom-up sensations. Imagined sensorimotor contingencies would generate prediction errors where they fail to align with actual sensory observations, which would influence conscious experiences if they reach hierarchically higher levels of cortex with rich-club connectivity [104,170]. These subnetworks are notable in having both high centrality and high reciprocal (or re-entrant) connectivity, which have been suggested to support “dynamic cores” of mutually-sustaining activation patterns [6,171], so implementing “global workspaces” [172] capable of both integrating and differentiating [173] multiple aspects of phenomena with sufficient spatiotemporal and causal organization for coherent conscious modeling [15,73,74].

While the account of conscious agency presented here is radically embodied, it parts ways with more radically enactivist “extended mind” interpretations of predictive processing [174]. According to radical enactivist interpretations of active inference, subjective experience is the entailment of an implicit model represented by the *entire system* of hierarchical relations within an organism’s brain, body, and environment. However, I have suggested that processes only contribute to consciousness to the degree they couple with dynamic cores of neural activity on timescales at which information is integrated into particular large-scale meta-stable states [73,74], with coherence enhanced by mechanisms for stabilizing and coordinating synchronous activity [175,176]. While minds are certainly extended [177,178], consciousness may be a more spatiotemporally limited phenomenon.

Dynamic cores of consciousness may play another central role in Bayesian brains as sources of robust and (meta-)stable predictions. Conscious driving of neural dynamics allows for several properties that would not be possible without centralized control processes. To the degree widespread availability of information—often taking the form of embodied simulation—allows for coupling with linguistic production systems and their combinatorial and recursive generative potential, this would vastly increase the stability, complexity, and flexibility of active inference. To the degree these expanded abilities allow for inferring temporally-extended events, they may provide bases for constructing abstract self-models and a new kind of symbolic order [13,179]. Under this regime of conscious symbolism, a new kind of dynamic core becomes possible as world models with extended causal unfoldings and structuring by abstract knowledge. Such generalized dynamic cores would be constituted by systems of mutually sustaining predictions, whose robustness would increase when intersecting predictions provide synergistically greater inferential power when combined (e.g., converging lines of evidence).

I propose *embodied self-models (ESMs)* as constituting self-sustaining robust inferential cores at multiple levels. At lower levels of abstraction, minimal ESMs [180] correspond to body maps organized according to 1st-person perspectival reference frames. At higher levels of abstraction, more elaborate ESMs correspond to 3rd-person perspective body maps and schemas. These 1st- and 3rd-person perspectival ESMs both develop in inter-subjective social contexts, potentially via the internalization of 2nd-person perspectives [181] and mirroring with (and by) others [182,183]. Essential aspects of core selfhood—with both embodied and symbolic objectified characteristics—may involve a kind of internal ‘mirroring’ of 1st- with 3rd-person ESMs, so establishing linkages of effective connectivity for advanced self-modeling (Cf. mirror self-recognition as test of sentience) and self-control. Through experience, these various ESMs become associatively linked to each other as a control heterarchy governed by diverse modes of selfhood at varying levels of abstraction.

Neural populations capable of realizing these various self-processes will also develop reciprocal connections with inferior frontal and temporal hierarchies over phonological action-perception cycles, grounded in respective outputs to the vocal apparatus and inputs to hearing. These functional linkages would provide bases for semantic understanding based on syntactic grammar, which may allow for thought as inner-speech as previously described. From this radically embodied perspective, linguistic thought is a kind of motor skill, which partially renders declarative knowledge as a special case of procedural memory. These symbolic capacities afford more complex modes of organization, where ESMs take the form of narrative-enhanced selves [111,156] with nearly unbounded semiotic potential [184,185,186], including multilevel interpersonal coupling [187,188], participatory sense making and shared intentionality [189,190], and structuring experience by abstract meanings [121,148,191].

I suggest we may interpret “dynamic cores” game-theoretically [192], and extend this concept to emergent patterns structuring minds across all levels. Under the Free Energy Principle, all persisting forms necessarily minimize prediction error, and as patterns vie for promoting their existence, these interactions would constitute a kind of game with both cooperative and competitive characteristics. A ‘core’ would be established whenever a set of predictions becomes sufficiently stable such that it is capable of functioning as a kind of dominant paradigm [193] in belief space. This core property could be obtained because of a kind of faithful correspondence between model and world, or simply because it arises early in development and so structures subsequent modeling (whether accurate or not). Embodied selfhood is a good candidate for a generalized core in providing parsimonious modeling of correlated activity between heterogeneous sensations, whether interoceptive, proprioceptive, or exteroceptive [75]. I suggest ESMs provide such powerful explanations for experience that they form a necessary scaffolding for all other aspects of mind, with different aspects of selfhood being understood as kinds of extended embodiment [194,195,196,197], ranging from material possessions [198] to social roles, and other more abstract senses of self and ownership [111]. From this view, psychological development would be reframed in terms of preserving and adapting various core patterns—in neo-Piagetian terms, assimilation and accommodation—so allowing minds to bootstrap themselves towards increasingly rarefied states of complexity.

Among these developmental milestones, perhaps the most significant major transition is acquiring capacities for self-awareness [199]. As suggested above with respect to the potential importance of mirroring, such self-models may develop via the internalization of social interactions involving various forms of intersubjective inference. While the richness of selfhood ought not be reduced to any given mechanism, focusing on action-perception cycles illuminates ways that various neural systems may contribute to the construction (and control) of different objectified self-representations. Given sufficient experience, imagined actions from 1st-person reference frames will be accompanied by auto-associative linkages to perceptions of similar actions from other points of view. These various viewpoints become ‘encoded’ by ventral visual stream neuronal ensembles, which can become consciously accessible via posterior medial cortices [73,74]. Conscious 3rd-person self-representations afford additional forms of modeling/control and navigation of complex contingencies, such as imagining multistep plans, potentially accompanied by visualizations of moving through spatialized time. [Speculatively, this sort of perspectival cross-mapping may have been facilitated by the evolutionary elaboration of white matter tracts connecting dorsal and ventral cortical hierarchies [200,201].

Objectified selfhood represents a major transition in evolution, indicating a movement from 1st-to 2nd-order cybernetics, wherein agents become capable of using processes of self-regulation to recursively model themselves as goal-seeking self-regulating feedback systems [202]. Thus, a radically embodied perspective may help us to understand not only the micromechanics of intentional goal-oriented behavior, but also the nature of self-consciousness and potentially uniquely human forms of agency. This constructed selfhood with metacognitive capacities via mental actions also suggests ways that compromised mechanisms of agency would contribute to varying forms of maladaptive functioning and psychopathology [203]. This constructed selfhood also suggests means by which pathological self-processes could be updated, potentially via the intentionally-directed attention towards somatic states described above as a proto-model of meditative practices [166,167].

### 4.2. Embodied Self-Models (ESMs) as Cores of Consciousness

#### 4.2.1. The Origins of ESMs

**Figure 6:**
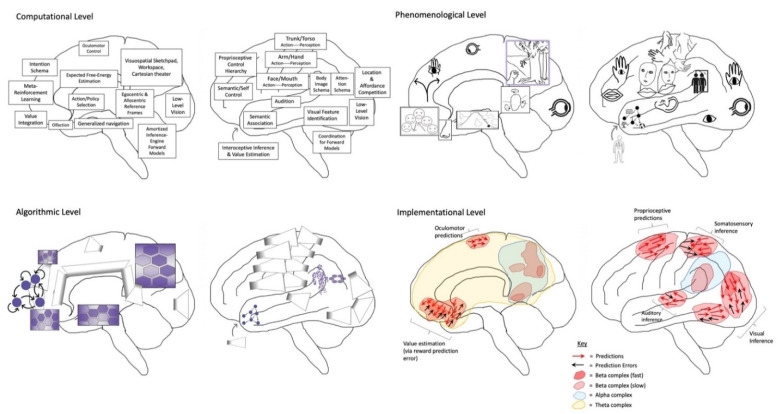
Depiction of the human brain in terms of entailed aspects of experience (i.e., phenomenology), as well as computational (or functional), algorithmic, and implementational levels of analysis [2,74]. A phenomenological level is specified to provide mappings between consciousness and these complementary/supervenient levels of analysis. Modal depictions connotate the radically embodied nature of mind, but not all images are meant to indicate conscious experiences. Phenomenal consciousness may solely be generated by hierarchies centered on posterior medial cortex, supramarginal gyrus, and angular gyrus as respective visuospatial (cf. consciousness as projective geometric modeling) [181,205], somatic (cf. grounded cognition and intermediate level theory) [3,206,207], and intentional/attentional phenomenology (cf. Attention Schema Theory) [118]. Computationally, various brain functions are identified according to particular modal aspects, either with respect to generating perception (both unconscious and conscious) or action (both unconscious and potentially conscious, via posterior generative models). [Note: Action selection can also occur via affordance competition in posterior cortices [94], and frontal generative models could be interpreted as a kind of forward-looking (unconscious) perception, made conscious as imaginings via parameterizing the inversion of posterior generative models.] On the algorithmic level, these functions are mapped onto variants of machine learning architectures—e.g., autoencoders and generative adversarial networks, graph neural networks (GNNs), recurrent reservoirs and liquid state machines—organized according to potential realization by neural systems. GNN-structured latent spaces are suggested as a potentially important architectural principle [208], largely due to efficiency for emulating physical processes [209,210,211]. Hexagonally-organized grid graph GNNs are depicted in posterior medial cortices as contributing to quasi-Cartesian spatial modeling (and potentially experience) [212,213], as well as in dorsomedial, and ventromedial prefrontal cortices for agentic control. Neuroimaging evidence suggests these grids may be dynamically coupled in various ways [214], contributing to higher-order cognition as a kind of navigation/search process through generalized space [215,216,217]. A further GNN is speculatively adduced to reside in supramarginal gyrus as a mesh grid placed on top of a transformed representation of the primary sensorimotor homunculus (cf. body image/schema for the sake of efficient motor control/inference). This quasi-homuncular GNN may have some scaled correspondence to embodiment as felt from within, potentially morphed/re-represented to better correspond with externally viewed embodiments (potentially both resulting from and enabling “mirroring” with other agents for coordination and inference) [39]. Speculatively, this partial translation into a quasi-Cartesian reference frame may provide more effective couplings (or information-sharing) with semi-topographically organized representations in posterior medial cortices. Angular gyrus is depicted as containing a ring-shaped GNN to reflect a further level of abstraction and hierarchical control over action-oriented body schemas—which may potentially mediate coherent functional couplings between the “lived body” and the “mind’s eye”—functionally entailing vectors/tensors over attentional (and potentially intentional) processes [218]. [Note: The language of predictive processing provides bridges between implementational and computational (and also phenomenological) levels, but descriptions such as vector fields and attracting manifolds could have alternatively been used to remain agnostic as to which implicit algorithms might be entailed by physical dynamics.] On the implementational level, biological realizations of algorithmic processes are depicted as corresponding to flows of activity and interactions between neuronal populations, canalized by the formation of metastable synchronous complexes (i.e., “self-organizing harmonic modes” [73]). [Note: The other models discussed in this manuscript do not depend on the accuracy of these putative mappings, nor the hypothesized mechanisms of centralized homunculi and “Cartesian theaters” with semi-topographic correspondences with phenomenology.].

To summarize, ESMs may form foundational cores and scaffolding for numerous and varied mental processes, ranging from the handling of under-constrained inference spaces to the intentional control of attention, imagination, and action. ESMs are both body maps and cybernetic control hierarchies, constituted by action-perception cycles grounded in skeletal muscle and associated perceptual efferents (Figure 6). As described above, the centrality of ESMs is expected based on early experiences [84,204] in which bodies provide learning curriculums wherein possibilities are fruitfully constrained [38], so allowing organisms to bootstrap their ways toward handling increasingly challenging modeling spaces within zones of proximal development [120]. With respect to the challenge of constructing robust causal world models—both enabling and possibly entailing conscious experiences [73,74]—the combinatorics of unconstrained inference spaces may only be surmountable via the inductive biases afforded by embodied learning. This fundamentally somatic developmental legacy suggests a radical perspective in which ESMs form a semi-centralized scaffolding for all intentional (and many unintentional) mental processes, grounding abstract symbolic thought in mental simulation and metaphorical extension from initial embodied experiences [47,48].

As described above, ESMs provide means by which action selection can be influenced via iterated comparisons of sensed and imagined sensorimotor states, with much complex planning achieved through backward chaining from goals, implemented via predictive coding mechanisms. Intentions (as self-annihilating free energy gradients) are proposed to function as systemic causes over neural dynamics, arising through interactions between beliefs and desires as counterfactual predominantly-interoceptive beliefs. Additionally, neuronal ensembles underlying ESMs—and the intermediate level representations they support [3,206,207]—may be positioned as centrally located, richly connected nodes in generative neural networks. On account of embodiment being functionally linked to most causes of sensory observations, coherent organization between ESM nodes would contribute to small-world connectivity, so enhancing message-passing potential, so enhancing capacity for informational integration. Thus, in addition to constituting the core of most mental processes, ESMs would be at the center of dynamic cores of neural activity [73,74,219], generating high degrees of integrated information [173,220] and instantiating communication backbones for global workspaces [221,222].

With respect to this hypothesis of workspace dynamics via ESMs, it is notable that periods of high and low modularity most strongly vary based on degrees of activity within sensorimotor and visual networks [223], potentially suggesting pivotal roles for these systems with respect to large-scale cognitive cycles [224]. Sensorimotor networks constitute the most extensive resting state component, involving 27% of overall grey matter [225]. Even more, these somatic networks establish a core of functional connectivity [226], with high degrees of overlap and coupled activity with other functional networks, including the default mode network, thus potentially linking conscious workspace dynamics to selfhood on multiple levels [227,228,229,230].

#### 4.2.2. Phenomenal Binding via ESMs

High degrees of mutual information across ESMs may enhance capacities for self-organized synchrony and inferential stability [204]. Indeed, the early emergence (with respect to both ontogeny and phylogeny) of body-centered neural responses suggests they may be foundational for extra-bodily forms of perceptual inference [91,231]. In terms of developmental primacy, studies of zebra fish demonstrate that spinal motor-neurons begin a stereotyped process of establishing global synchronization dynamics, beginning with the reliable enabling of increasing degrees of synchronous local activity [232], followed by larger-scale integration (or self-organization) into well-defined oscillatory modes as critical thresholds are surpassed [233]. High degrees of integrative capacity via body maps may potentially help to explain the remarkable capacities of nervous systems to reconfigure themselves for both good (e.g., recovery after injury) and ill (e.g., phantom limb syndrome) [194,234,235].

Theoretically, ESMs may transfer some of their synchronous (and inferential) stability to non-body representations (e.g., external objects) when functionally coupled. This coupling could be realized by the driving of simulated (and sometimes overtly enacted) actions by reactive dispositions and perceived affordances [94,95]. Affordance relations must have physical bases in neuronal ensembles—even if highly dynamic and context-sensitive—constituted by representations of action-perception cycles, grounded in bodily effectors and sensors. If non-body representations are auto-associatively linked to ESMs via affordance relations [71], then synchronous dynamics within ESMs could transitively entrain neural ensembles for non-body representations, so increasing their perceptual stability. With relation to perceptual binding, specific affordances could contribute to specific patterns of synchrony, so instantiating specific networks of integration, which in some instances may entail phenomenal experience and potentially conscious access. [Note: The other models discussed in this manuscript do not depend on the accuracy of this hypothesis of *phenomenal binding via ESMs*.]

Mechanistically, traveling waves [236,237,238] from ESMs could form major points of nucleation for the formation of large-scale meta-stable rhythmic attractors [229,239,240,241,242]. Such self-organizing harmonic modes likely have multiple functional significances within nervous systems [73,74], including the ability to coordinate large-scale patterns of brain activity. This model of resonant binding via simulated embodied engagements further suggests that partially-expressed motor predictions with specific affordance linkages could be used for attentional selection over particular objects. From this point of view, enactivist discussion of “optimal grips” [89] may potentially indicate a foundational mechanism by which conscious access is realized via fictitious motor commands. Consistent with linguistic use, there may be a surprisingly (or perhaps intuitively) meaningful sense in which we “hold” objects in mind with attention (as partially-expressed motor predictions), potentially providing a neurocomputational understanding for the word “concept” in terms of its etymological origins (i.e., “*to grasp*”).

ESMs are proposed to form cores of consciousness as dominant sources of integrated effective connectivity across the entire brain, facilitating coherent perception and action. ESM-grounded consciousness would not only imbue all percepts with the affordance potential of sensorimotor contingencies [169], but also the previously discussed sense of “presence” as perceptual depth from counterfactual richness [154,243], so illuminating fundamental aspects of phenomenology. If this model of virtual enactive binding and manipulation of percepts is accurate, then we may possess yet another account of the roles of frontal lobes with respect to global workspace dynamics and higher-order consciousness. While posterior cortices may generate conscious experiences of space [73,74,212], frontal cortices may provide bases for cognitive ‘work’ in the form of the stabilization and manipulation of percepts within these mental spaces (Figure 6).

This radically embodied view has received some support from findings in which motor information heavily influences neural signaling in almost every modality [244,245,246]. Notably, parietal cortex provides sources of both high-level body representations as well as spatial awareness, with damage not only resulting in anosognosia and alien limb syndromes, but also hemi-spatial neglect [218]. There is also a counter-intuitive finding in which the spatial extent of neglect symptoms are extended via providing a reach-extending tool for the hand corresponding to the affected side of space [195,247]. Speculatively, affordance-based bindings via ESMs may potentially provide a partial explanation for this surprising phenomenon, in that neglect symptoms could result from coupling with ESMs whose coherent (synchronous and inferential) dynamics have been compromised. Resonant coupling between percepts and ESMs may also help explain how external objects—potentially including other agents [248]—may become incorporated into body maps [249], with synchronous motions helping to establish expansion/binding. These fundamentally-embodied bases for phenomenality could also be (indirectly) evidenced by impaired memory with out-of-body states [250], and superior memory accompanying 1st-person points of view [251].

Recent work from Graziano and colleagues may provide support for this model of perceptual binding via ESM-based affordances. In Attention Schema Theory (AST) [118,252], conscious awareness is thought to correspond to reduced-dimensionality schematic modeling of attention, providing an informational object that is simpler to predict and control, relative to that which is modeled. The sketch-like nature of attention schemas makes them unamenable for clear introspection, so contributing to an anomalous inference wherein awareness is implicitly (and sometimes explicitly) viewed as a fluid-like physical substance that comes out of a person’s eyes and reaches out into the world, so contributing to the “extramission myth of visual perception.” Researchers from Graziano’s lab [253] found evidence for an intriguing phenomenon in which seeing another person’s gaze appeared to result in inferences of force-transfer towards an unstable object. This finding is consistent with the ESM-based model of perceptual binding described above, although variations on the experiment might provide an opportunity to uniquely test the hypotheses proposed here. According to the “eye beams” model of AST, implicit forces associated with gaze should always be a push—due to the implicit anomalous inference that awareness is like a fluid that can be emitted—causing the object to be more likely to fall away from observers. However, according to the model of phenomenal binding via ESMs, the force would either push or pull, depending on associated affordances, and possibly affective states.

In AST, conscious awareness is suggested to be the phenomenal entailment of attention schemas and the representations they bias. In the radically-embodied view described here, attention schemas would represent upper levels of control hierarchies over action-oriented 1st-person body schemas [118], or ESMs as action-perception hierarchies distributed across frontal and parietal cortices (Figure 6). The neuropsychological literature provides some support for this idea, with frontal and parietal lesions both contributing to neglect symptoms [254]. The centrality of the temporoparietal junction (TPJ) for conscious awareness in AST [218] points to possible functional overlaps between networks establishing embodied selfhood and conscious awareness. Notably, TPJ disruptions can result in perceptual anomalies such as out-of-body experiences and body-transfer illusions [255,256]. Associations between mental state inference [257] and overlapping representations for self and other in the TPJ (and dorsomedial PFC) provides further support for social bootstrapping of objectified selfhood described above. High-level action-oriented body maps may be indispensable for attempting to infer mental states and intentions, whether through “mirroring” or perspective-taking via attention schemas shared between self and others [118,121,190,258,259,260]. Thus, conscious access might not only depend on radically embodied minds, but may also fundamentally involve intersubjective modeling [187,188,261].

#### 4.2.3. Varieties of ESMs

*“We suggest that a useful conceptual space for a notion of the homunculus may be located at the nexus between those many parallel processes that the brain is constantly engaged in, and the input from other people, of top-top interactions. In this understanding, the role of a putative homunculus becomes one of a dual gatekeeper: On one hand, between those many parallel processes and the attended few, on the other hand be-tween one mind and another... [T]he feeling of control and consistency may indeed seem illusionary from an outside perspective. However, from the inside perspective of the individual, it appears to be a very important anchor point both for action and perception. If we did not have the experience of this inner homunculus that is in control of our actions, our sense of self would dissolve into the culture that surrounds us.”*

—Andreas Roepstorff and Chris Frith [12]

In this account, ESMs function as sources of maximal model evidence in FEP-AI [75], complexes of integrated information [173,220], and backbones for global workspaces [129]. This view of consciousness and agency centered on ESMs is consistent with both the information closure [262] and intermediate-level [207] theories of consciousness. Intermediate levels of abstraction afford embodied simulation [3,206,263], wherein action-perception cycles enable cybernetic sense-making and grounded cognition. Indeed, cybernetic grounding via ESMs could partially help in explaining why consciousness may arise “only at the personal level” [264].

ESMs are composed of multilayer control hierarchies at varying levels of abstraction, ranging from 1st-person interfaces, i.e., the “lived body” [43,181], to 3rd-person body schemas capable of acting as symbolic and self-reflexive intentional controllers. The singular embodied self and models of selfhood as a “center of narrative gravity” [228,265] imply multiple roles for unified embodied representations as high-level control processes, organized according to multiple perspectival reference frames. The complexity and specificity of these models of self and world are greatly expanded by the combinatorial and recursive properties of language [156,179], including temporal extension and stabilization via organization into diachronic narratives [184]. While consciousness may not depend on language for its realization, linguistic capacities may have profound impacts on the evolution and development of conscious awareness, selfhood, and agency.

Multilevel integration via selfhood may represent a necessary condition for perceptual coherence by providing binding from core embodiment. Similarly, in line with renormalization group theory and the enslaving principle of synergetics [266,267], the ability of self-processes to stably persist through time provides reduced-dimensionality attracting center manifolds capable of bringing order to—or generating selective pressures over—faster dynamics at lower levels of organization. A slower, larger, and more centrally positioned set of dynamics has asymmetric potential to entrain (or enslave) faster and more fleeting processes, which will be relatively less likely to generate cohesive influences due to their transient character. Self-processes can be viewed as sources of highly coherent meso- and macro-scale vectors—or effective field theories [268]—over biophysical dynamics, allowing systems to explore state spaces in ways that would be unlikely without centralized integrative structures.

Selves provide spatial and temporal structure for complex sequences at multiple levels of abstraction, including symbolically. Such abstract integrative structures are referred to as “narratives” [184,269,270], for which it is no coincidence that such modes of organization facilitate learning, and where the act of telling and listening to stories is a human universal [271,272]. In terms of control systems, narratives allow for coherent stabilization of evolving conceptual structures in ways that provide multilevel syntax, so affording planning on multiple temporal and spatial scales. Narratives with multiscale organization provide one of the best ways to model and control such extended processes, including with respect to the narrativizing processes that both help to generate and are governed by self-models. In these ways, agentic selfhood is a story that becomes (more or less) true with the telling/enacting.

At their most basic, selves are constituted by models of embodiment and embedding within the external environment. At their most complex and abstract [273]—returning to the evolutionary game-theoretic considerations described above with respect to generalized dynamic cores—selves are patterns with which agent-like-systems are most consistently identified, where agentic systems are construed according to a kind of projected revisionist victor’s history [14,265,274], wherein victors are constituted by dominating coalitions of patterns, bound together by evolving interactions between habits, narratives, and specific niches constructed by agents. Inter-temporally coherent belief-desire coalitions more consistently achieve higher value [275,276], and so tend to be reinforced, and so tend to dominate persona evolution [60]. Shared narratives co-evolving with these pattern coalitions [271,277,278] are shaped by repeated games both within [279,280,281] and between individuals [121,269,282]. Although self-processes may become extremely complex (and abstract) in these ways, in all cases such generative models both originate from and must continually deal with the constraints and affordances of their radically embodied nature.

### 4.3. Free Energy; Will Power; Free Will

The self-sustaining stability and predictive power of multilevel dynamic cores constitute free energy reservoirs [73,283], capable of enslaving hierarchically lower levels, and so driving overall systems towards novel (and surprising) regions of state space predicted in imagination. By this predictive processing model, will power is proportional to the strength with which an agent predict/imagine actions for desired states in the face of obstacles to goal attainment. The embodied attention mechanisms described above provide organism-centered (and potentially more intuitively controllable) means of boosting the predictive power of specific representations. These distributed high-level controllers necessarily grow from histories of predictive homeostatic regulation (i.e., allostasis via active interoception), largely centered around control hierarchies spanning insular and cingulate cortices [284,285], which influence neuromodulatory value signals through direct and indirect connections to hypothalamic and brainstem nuclei [286,287].

The radically embodied proposal presented here is that all self-control processes have their origins in controlling skeletal muscle, both via multilevel shared mechanisms, as well as via metaphorical extension from experiences with movement [288]. To the extent these regulating dynamics depend on particular neuroanatomical hubs, conscious willing constitutes a limited resource to the degree that sustained activity results in degradation of efficient predictions. This is consistent with rest periods being required to avoid “ego depletion” [289], possibly via mechanisms involving slow wave activity and synaptic downscaling within these hubs [290,291,292,293]. Based on the models described above, these executive resources would heavily depend on networks utilized for simulating actions of varying degrees of complexity, with fictitious foveations and virtual motoric manipulations likely being especially impactful (Table 2). Dorsomedial and dorsolateral prefrontal cortices provide higher-order control over frontal eye fields and pre-supplementary motor areas [74] (Figure 6), which have both been associated with attention and working memory [125,294,295]. Strong evidence for these models would be obtained if executive failure (and recovery) were reliably indexed by local increases (and subsequent decreases with rest) in slow oscillations, as well as if stimulation [296] applied to these areas—or perhaps other integrative networks [297]—was found to increase self-control and promote repletion during rest intervals.

However, even without exhausting limited (but flexible) neural resources, sustained willing may be preemptively curtailed based on explicit and implicit predictive models of ongoing dynamics [298]. In the context of goal-pursuit, emotional states reflect a balance between inferred benefits and costs associated with various goals [162], including estimates of opportunity costs, which have both direct and indirect effects on motivating/energizing (or inhibiting) behavior. These proactive regulatory mechanisms largely stem from insular and cingulate cortices acting as predictive homeostatic (i.e., allostatic) control systems, as well as from additional converging inputs to neuromodulatory processes (e.g., dopaminergic nuclei of the brainstem), so influencing thresholds for neuronal activity cascades and subsequent overt actions.

Other self-control limitations may be difficult to describe in terms of specific neural systems, but may instead emerge from heterogeneous predictions regarding value attainment associated with goal-pursuit. For example, it may be the case that self-processes become more causally efficacious in minds to the extent that they are predicted to be causally efficacious in the world. In these ways, there could potentially be bidirectional relationships between willpower, situation-specific self-efficacy, and even global self-esteem.

A radically embodied cybernetic Bayesian brain suggests multiple mechanisms by which we can be said to have (within limits) the “varieties of free will worth having” [299]. While debates regarding the ontological status of free will may not be definitively resolved in this manuscript, we have shown that intentions—as conjoined beliefs and desires—can function causally in their ability to act as coherently stable predictions. To the extent these predictions can be maintained in the face of discrepant observations, these sources of control energy will drive overall dynamics. Thus, conscious mental states are not only “real patterns” [300] because of their significance for experiencing subjects, but also because consciously ‘held’ intentions may meaningfully contribute to cognitive (and potentially thermodynamic) work cycles [13,73,301,302].

### 4.4. Mental Causation

This mental causation could be similarly described in the language of generalized Darwinism [279], with preferences functioning causally within minds in the same ways that selective pressures [303,304,305,306] are causal within evolutionary systems [17]. More enduring preferences can be viewed as ultimate-level causes that select for the development of context-specific proximate-level choices [307]. We may further think of motor control via hierarchical predictive processing in terms of a hierarchy of selection processes. In this view of action selection as a kind of natural selection, hierarchically lower levels provide specific adaptations for realizing hierarchically-higher selective pressures, the totality of which constitute the overall direction of ‘will’ in any given moment. On longer timescales, histories of experience change beliefs and desires, so providing another way in which preferences act as (recursively self-modifying) causes for minds as multilevel evolutionary systems.

Intriguingly, the concept of ‘pressure’ and the ability of free energy gradients to drive work may be isomorphic when considered in the contexts of Bayesian model selection, natural selection, and thermodynamics [308,309,310]. Although post hoc confabulation occurs [311,312], in many cases the driving of behavior via intentions may be viewed as (formally) similar to the powering of engines via controlled explosions. Further, in the gauge-theoretic framing of the Free Energy Principle [158], precision weighting is formally understood as a kind of (symmetry-preserving) force in precisely the same sense as gravity is a force resulting from the deformation of spacetime. Therefore, desires and willpower may be forces in every meaningful sense of the words ‘power’ and ‘force.’

Can things as seemingly ephemeral and abstract as beliefs and desires have causal powers in the same senses as in physics? Perhaps this is just an exercise in semantic games, playing with metaphors and words to avoid the obvious and inevitable conclusion: the only real causation is physical, and any other sense of cause is mere expediency, representing an approximate attempt at explaining and predicting events whose underlying reality is too complexly determined and difficult for us to measure and understand. Perhaps. Yet it is also the case that ‘causes,’ ‘powers,’ and ‘forces’ are themselves just words, or metaphors, or models for the phenomena they attempt to represent in compressed form, and where they would lack explanatory or predictive utility without dimensionality-reducing approximations [48,59]. Occasionally we need to remember that the meanings of words are determined by our interacting minds, wherein they are always (without exceptions) mere expediencies—even if this expedience also affords the evolution of civilizations and the technologies upon which we depend for life [271]. The word ‘cause’ is mostly lacking in physics, as most physicists have no need of singling out specific things in order to explain or predict particular events [268]. Master equations of dynamics may be specified such as Hamiltonians and Lagrangians, from which system-evolution flows deterministically, but the notion of causation is not found in such descriptions. The absence of causal notions in physics makes sense in light of physical laws being symmetric with respect to time, and where time may be an emergent local description, rather than a fundamental principle of the universe [313]. Even ‘force’ has been deflated in fundamental physics, and instead replaced with “fictitious force” in conceptualizations such as the gauge constructions underlying relativity and other field theories [158]. On account of the conceptual elegance of these theories, many physicists no longer talk about “fictitious” forces, since it could be argued that there are no other kinds.

Perhaps even more fundamentally [314], if we trace the genealogy of these concepts, and so understand the radically somatic origins of minds, then we might discover our notions of cause and force were initially derived via metaphorical extension from embodied experiences of volitional control [16,48,59,288]. This is not to say that it is permissible to commit a genealogical fallacy and reduce the realities of these concepts to their beginnings. Formal accounts of causation have been provided in terms of operations over graphical models involving manipulations of dependencies via counterfactual interventions [119]. However, such handlings require commitment to a given ontology (i.e., carving up a domain into particular kinds), and do not support reducing processes to more fine-grained dynamics where higher-level properties are undefined. Even if temporality is found to be fundamental (rather than emergent) in ways that afford causal modeling over some ‘true atomism,’ reductive explanations would still not be of an eliminative variety. Eliminative perspectives on emergent phenomena (such as intentionality) may be literally meaningless and nonsensical, in that they violate the rules of logical reasoning whereby sense-making is made possible.

Alternatively framed, intentions (as conjoined beliefs and desires) could be viewed as kinds of “effective field theories” over psychodynamics and behavior [268], affording maximally powerful ways of explaining and predicting events whose underlying statistics afford (and demand) coarse-graining [315] in ways that give rise to new ontologies. In these ways, beliefs and desires are as real as any-‘thing’ [64], even if there is a wider (but nonetheless constrained) range of plausibly useful interpretations, relative to ‘things’ like particles. However, a proper understanding of the formal properties underlying these more rarefied emergent phenomena—as generalized evolution [308,309]—may be shared among all similarly configured physical systems. Therefore, our intentions really are sources of cause, power, and force in every meaningful sense of these words. Our intentions are real patterns [300], and so are we.

## 5. Neurophenomenology of Agency

### 5.1. Implications for Theories of Consciousness: Somatically-Grounded World Models, Experiential Richness, and Grand Illusions

*“For my part, when I enter most intimately into what I call myself, I always stumble on some particular perception or other, of heat or cold, light or shade, love or hatred, pain or pleasure. I never can catch myself at any time without a perception, and never can observe any thing but the perception. When my perceptions are remov’d for any time, as by sound sleep; so long am I insensible of myself, and may truly be said not to exist. And were all my perceptions remov’d by death, and cou’d I neither think, nor feel, nor see, nor love, nor hate after the dissolution of my body, I shou’d be entirely annihilated, nor do I conceive what is farther requisite to make me a perfect non-entity... But setting aside some metaphysicians of this kind, I may venture to affirm of the rest of mankind, that they are nothing but a bundle or collection of different perceptions, which succeed each other with an inconceivable rapidity, and are in a perpetual flux and movement.”*

—David Hume [316]

As described in previous work [73,74], consciousness can be understood as the capacity of minds to support global workspaces [317], defined by dynamic cores of competing and cooperating patterns [171,219], which depend on—but are not identical to—a system’s integrated information [173,220]. However, the deeply embodied perspective described here suggests that for systems to be conscious, integrated information must apply to representations with experience-grounded meanings. These representations need not be explicitly defined symbols, but their semiotic content could be entailed in a cybernetic manner via the coordination of action-perception cycles. A neuronal complex could have an arbitrarily high amount of integrated information, but it may not be conscious unless it also refers to patterns external to the system. Capacity for consciousness may be proportional to (but not necessarily defined by) integrated information from dynamics with representational content. One of the primary adaptive advantages of consciousness may be enabling representations—computationally realized a balance of integrated and differentiated dynamics—that evolve on timescales roughly proportional to events in the world that systems attempt to control, so enabling cybernetically-grounded meaning making. [For perceiving dynamics on spatiotemporal scales where more direct coupling is infeasible, we may require (embodied) metaphor, such as may be used in the spatialization of time [318,319].] By this view, informational objects in “qualia-space” [320] would have phenomenal content by virtue of being isomorphic with probability distributions of generative models over bodily sensoriums for systems that evolve-develop through interactions with environments in which they are embedded. Thus, a radically embodied perspective may be essential for explaining the circumstances in which integrated information does or does not imply conscious experience.

The models presented here are also consistent with Higher-Order-Thought [321] theories emphasizing the importance of the frontal lobes in conscious awareness and intentionality, whose functional connectivity with parietal (and temporal) regions may be crucial for stabilizing representational content [6,11]. Anterior portions of prefrontal cortex may be particularly pivotal/central in establishing small-world connectivity for the entire brain [170], so affording large-scale (flexible) availability of information. While this area may be particularly well-connected across primate species [322], this connectomic hub may have been uniquely expanded in humans relative to non-human primates [179,323]. However, a radically embodied perspective suggests that prefrontal hubs may not merely establish global connectivity. Rather, these systems may specifically function as upper levels of hierarchies shaping dynamics via simulated actions and partially-expressed motor predictions (Figure 6), so providing a basis for intentional control. In this way, the frontal lobes as subserving “executive functions” may be something more than a ‘mere’ metaphor, but may also be an apt description of a quasi-homuncular hierarchical control architecture centered on body-centric agency. We may even want to go as far as recasting the notion of “access consciousness” [168] to depend on the kinds of fictitious mental acts described above for realizing meta-cognition and conceptual thought, understood as abstract motor skills, potentially involving resonant phenomenal binding via embodied self-models (ESMs).

As described in previous work [73,74], not only may there be something of a Cartesian theater, but percepts may be re(-)presented on/in this virtual reality screen (Figure 2, Figure 4, Figure 5 and Figure 6). Further, as described above, quasi-homuncular ESMs (as multimodal action-oriented body maps) would introspect the contents of these spaces with partially-expressed motor predictions, with simulated foveations—and other fictitious actions (Table 2)—providing sources of both (a) attentional “spotlights,” and (b) coherent vectors for intention and action. However, what is the extent of this unified field of experience? Do we actually fill in a full and rich simulated environment, or is this subjective experience some kind of “grand illusion”, where in fact we only fill in local aspects of the environment in an ad hoc fashion [8,324,325,326]? Rather than filling in a complete sensorium all at once, might we instead generate percepts reflecting the sensory acuity accompanying our actual sensorimotor engagements, which may be surprisingly limited (e.g., proportional to the narrow field of view afforded by the focal region of the retina)?

Phenomena such as invisible ocular blind spots suggest some perceptual filling occurs, and which is something the brain’s generative models may be well suited to provide [73,74]. However, the extent of this pattern completion remains unclear, and may be surprisingly sparse. For example, to what extent does the “visuospatial sketchpad” model of working memory actually involve a kind of internal sketching, potentially even involving the internalization of actual experiences with drawing [327]?

Indirect evidence for low-dimensional inner-sketching may be found in work in which similarities were observed between models of internal visual percepts and behavioral line drawings [328]. The authors note that such images can be traced back to Paleolithic hunters 40,000 years ago (with possibly earlier origins), suggesting that line drawings not only represent effective means of conveying meanings, but may also reveal functional principles of the visual system. While this particular study focused on predicting responses in the ventral stream, patterns of neural activity in the posterior medial cortex may be particularly important in having strong correspondences with visual consciousness (Figure 6). That is, feature hierarchies of the ventral stream may help to coordinate evolving spatiotemporal manifolds in posterior medial cortices as consciously accessible 2D sketchpads. Some support for this model is provided by a study in which attention and working memory indicated 2D mappings of the visual field [329]. Connections between this midline structure and upper levels of other sensory hierarchies further allow for the (partial) filling-in of multimodal somatosensory states, so providing bases for not just a Cartesian theater, but fully immersive virtual reality [49]. Even more, connections between these various modalities of experience with the hippocampal-entorhinal system could allow this somatic pattern completion to evolve according to trajectories through physical and abstract spaces, so providing a basis for episodic memory, novel imaginings, and planning (Figure 3 and Figure 4). With respect to the filling-in process, the specific contents of consciousness may depend on the specific degree to which representations from various sensory hierarchies are capable of coupling with large-scale meta-stable synchronous complexes on their temporal and spatial scales of formation [73,74].

While conscious experience may be “flat” [330] in terms of being of surprisingly low dimensionality, the functioning of consciousness within overall mental systems may also be deep. The multiply-determined contextual significances of reduced-dimensional processing is potentially reflected in nearly all languages converging on a common information transmission rate of ~39 bits/second [331]. Theoretically, the limited dimensionality of conscious processing may be a primary reason for this communicative bottleneck. However, the generative potential of consciousness and expressive power of language (with its “infinite use of finite means”) may nonetheless afford supra-astronomical semiotic capacities. Even if integrative dynamic cores and global workspaces have extremely limited capacities, they may nonetheless possess depth and powerful combinatorics via spanning levels both within and across hierarchically-organized systems, so constituting multiscale functional heterarchies. The temporally-extended nature of conscious processes [332,333] affords numerous and varied opportunities for shaping by complex unconscious dynamics, many of which can be given coherent organization by diverse—but capable of being integrated, to varying degrees—self- and world-modeling on multiple levels, whose richness is greatly expanded by narrative organization in the ways described above [184].

While some of the richness of consciousness may represent a “grand illusion”, in many ways this supposedly illusory phenomenon may function as if a rich and full field were always present by filling in details on an as-needed basis. Given this availability of relevant information, in addition to having many of the “varieties of free will worth wanting” [299], we have many of the varieties of conscious experience worth wanting as well. Consciousness would only appear to be “flat” if we fail to consider its nature(s) as a temporally-extended unfolding of generative processes [73,74,334]. Thus, the illusory nature of rich consciousness may itself be something of an illusion due to trying to model inherently time-dependent processes from an atemporal perspective, which would be prima facie inadequate for evaluating relevant phenomena. [Note: Deflations of deflationary accounts of selfhood may be arrived at in a similar fashion, including with respect to Buddhistic/Humean reductions of selfhood to non-self elements.]

### 5.2. Conscious and Unconscious Cores and Workspaces; Physical Substrates of Agency

Although a detailed handling is beyond the scope of the present discussion, a variety of methods may be useful for estimating subnetworks (e.g., giant components) contributing to consciousness [335,336,337], and perhaps agency. One intriguing study used k-core decomposition to track transitions from conscious to unconscious subliminal perceptual states [338]. Surprisingly, the most connected kernel and inner core of the conscious state remained functionally active when the brain transitioned to the subliminal-state. Not only may activity within the inner-most connectivity core of the brain be unconscious, but conscious access was lost by inactivating peripheral shells, potentially suggesting the importance of sensorimotor information for enabling coherent experience. These findings suggest that accessible consciousness might not be generated in the inner-most core, but at intermediate levels of hierarchical organization and abstraction [207,262], potentially involving the kinds of fictitious action-perception cycles described above with respect to meta-cognition and self-consciousness.

These findings could also potentially illuminate otherwise mysterious phenomena, including things like intuitive cognition [339], “tip-of-the tongue” effects, and even the roles of spontaneity in agency [314,340]. Some aspects of intuition and semi-conscious percepts may correspond to attractor dynamics accumulating in an (unconscious) inner-most core and outer shells and bypassing intermediate levels. Alternatively, in line with the “isolated qualia” model described above, information may be capable of driving action selection and conscious imaginings from networks supporting (consciously experienceable) embodied simulations—potentially the 1st shell out from the inner core—but without sufficient robustness to be stably introspectable.

While agency might typically depend on predictability for the sake of controllability, there may be ways in which overall control is enhanced by limitations of self-prediction:

- Avoiding excessive exploitation (at the expense of exploration) in action selection (broadly construed to include mental acts with respect to attention and working memory).
- A process for generating novel possibilities as a source of counterfactuals for causal reasoning and planning.
- Game theoretic considerations such as undermining the ability of rival agents to plan agonistic strategies, potentially even including “adversarial attacks” from the agent itself.

In these ways, somewhat paradoxically, agency may sometimes be enhanced by limiting the scope of intentional control.

Relatedly, intriguing work in artificial intelligence models the frontal pole as a recurrent neural network whose capacity for chaotic bifurcation enables flexible action selection and predictive learning [341,342]. Recurrent computational reservoirs have high potential for informational density due to the combinatorics of re-entrant connections, but more overtly hierarchical architectures have the advantages of discrete compositionality (so affording precise control) and robustness-via-modularity (so affording separable optimization). Cortical systems may leverage both of these capacities by placing a recurrent bifurcating nexus on top of a hierarchy of action-perception cycles with more linear dynamics [43] (Figure 6). The capacity of recurrent systems to exert recursive causal influences on themselves makes them chaotic systems with sensitivity to initial conditions. In these ways, upper regions of cortical control hierarchies may be occupied by processes that are inherently inaccessible to conscious modeling. Notably, these deepest portions of cortex are also newest with respect to both evolution and development [179], and have many of the properties we normally associate with personhood [143], including individuality [343,344], spontaneity, and autonomy [345].

If these models regarding the neural substrates of consciousness are accurate, then they may also help contextualize findings where agency appears to be missing. The *Libet experiment* [346] provides a particularly notable example of a supposed demonstration of non-agency, as the subjective experience of deciding to move was observed to emerge *after* predictive neural activity. Potential limitations of the paradigm notwithstanding [299,347,348], the question arises as to how conscious mental states could be causal, given that we expect causes to precede effects. Theoretically, reports regarding decisions to act occurring after predictive neural signals could be partially accounted for by effective connectivity between preparatory motor activity and largely unconscious inner cores.

If actions may be ‘decided’ by processes outside of conscious awareness, then is our sense of free will another grand illusion? Perhaps in some cases, but probably often not with respect to the “varieties of free will worth wanting” [299], as much meaningful executive control does not involve the generation of motor deployment events based on capricious whims. Such spontaneous acts might primarily be governed by stochastic activity within hierarchically lower levels, closer to primary modalities that have less access to richly connected networks where large-scale (consciously accessible and controllable) coordinated activity would tend to center [203]. Most actions do not occur as one-off events, but unfold within contexts involving conscious imagining and planning (Table 1, Figure 2), which can substantially drive overall neural dynamics. Similarly to the previously discussed case of the apparent flatness of consciousness and the supposed insubstantiality of selfhood, we may find ourselves denying the existence of “real patterns” [300] based on investigations that were ill-equipped to capture the relevant phenomena. In some senses we might identify agency (and personhood) with overall systems with both conscious and unconscious components. Such systems (and persons) may not be strongly shaped by consciousness in any given moment, yet could be significantly consciously shaped over time. Agency may be like the relationship between conductor and an orchestra, where conductors are neither omnipotent nor mere epiphenomena. Or to use the metaphor of the elephant and its rider: elephants with and without riders are very different “beast machines” [349].

### 5.3. Readiness Potentials and the Willingness to Act

Alternative explanations for Libet phenomena may be found in the Free Energy Principle and Active Inference (FEP-AI) framework [55], wherein brains are understood as cybernetic control systems that predictively model the world [59,60,61]. As previously described, within FEP-AI, support is accumulating for an associated process theory of hierarchical predictive processing (HPP) as a unified principle governing neural functioning [20,148,350]. In HPP, all brain areas generate top-down predictions over bottom-up inputs, where information is only passed upwards (as prediction error) if it fails to be predictively inhibited. Support for this common cortical algorithm is evidenced by theoretical considerations (e.g., efficiency), and is consistent with common architectural principles reflected throughout cortex [18]. HPP suggests both perception and action are inherently interrelated and fundamentally similar: perception minimizes prediction error via updating internal models, and action realizes this objective by updating world states to better match predictions. Action selection is understood as the (complementary) inverse of perception: perceptual hierarchies are updated via ascending prediction errors, and action hierarchies are updated via descending predictions [351]. Particular actions are selected as more complex/abstract predictions from higher areas cause cascades of more fine-grained lower-level predictions, ultimately driving motion via spinal motor pools and associated reflex arcs with skeletal muscles [72].

HPP (and FEP-AI more generally) may represent a “Rosetta stone” for neuroscience [71], allowing new interpretations of previously ambiguous phenomena, potentially including the nature of *readiness potentials (RPs)* associated with seemingly voluntary movement decisions [352]. This multilevel modeling framework could prove invaluable for investigating the functional significances of RPs and associated waveforms [353]. FEP-AI would understand these slowly-building potentials as evidence accumulation with respect to predictive models, accompanied by non-linear phase transitions in large-scale updating of implicit (and sometimes explicit) Bayesian beliefs over proprioceptive poses [73,74,106,334]. Through HPP mechanisms, these discretely updated predictions would constitute kinds of self-fulfilling prophecies when passed down cortical hierarchies with sufficient power to drive overt enaction.

**Figure 7:**
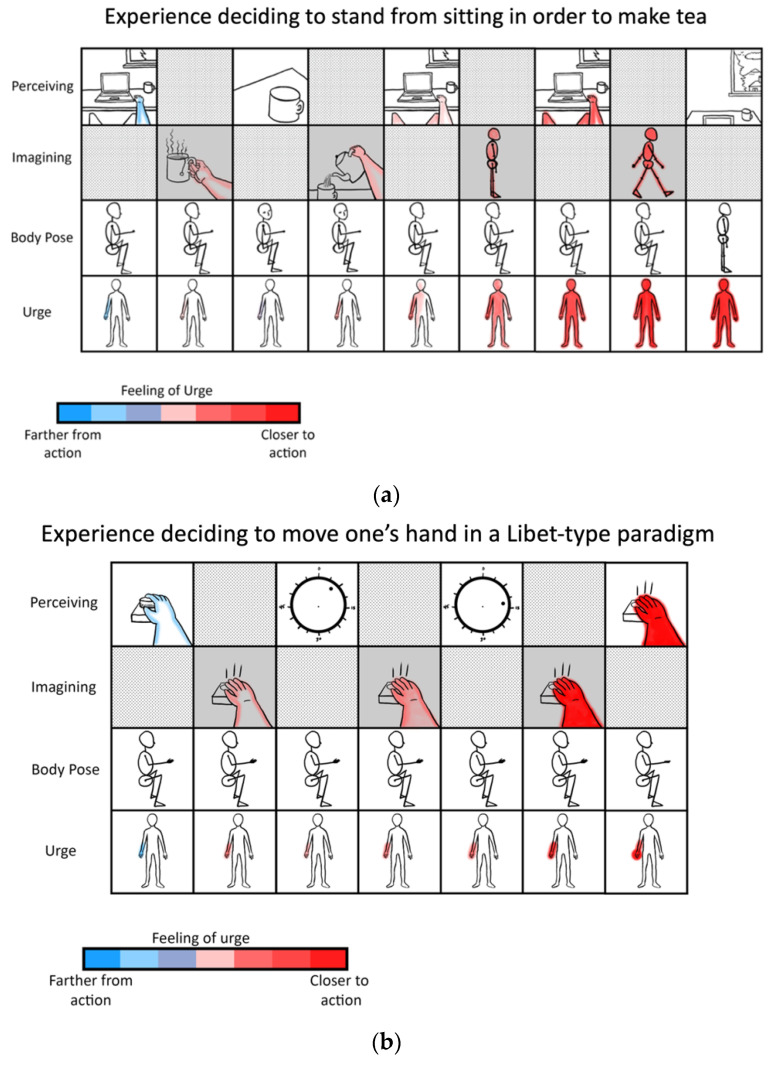
(**a**) Experience deciding to stand in order to make tea (see Table 1 and Figure 2, Figure 4 and Figure 5). The individual alternates between (1) perceiving sitting with an empty cup, and (2) imagining actions related to achieving the desired goal of obtaining tea. As the individual imagines (or rehearses) possible actions, feelings of urge accumulate across multimodal body maps, which peak accompanying the overt enaction of standing. (**b**) Experience deciding to move one’s hand in a Libet paradigm. The individual alternates between (1) perceiving one’s hand and the clock and (2) imagining button-pressing. As possible actions are imagined/rehearsed, feelings of “urge” accumulate across multimodal body maps, which peak accompanying the overt action of button-pressing.

**Figure 8:**
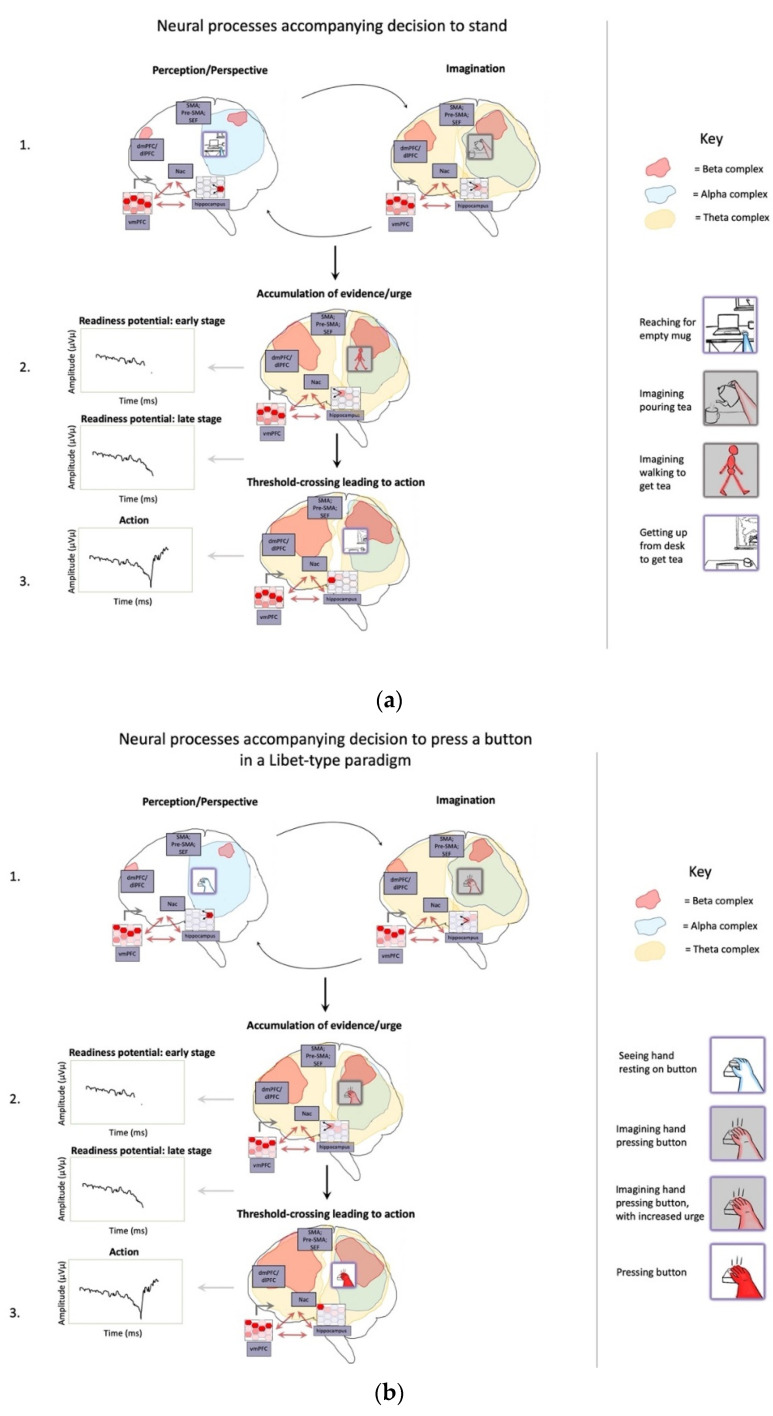
Readiness potential reflecting the accumulation of urge from simulated actions. (**a**) Neural processes accompanying decision to stand (see Figure 7a). (**b**) Neural processes accompanying decision to press a button in a Libet-type paradigm (see Figure 7b). Imaginative simulations (alpha oscillations, blue shading) are hippocampally orchestrated (Figure 4) via theta oscillations (yellow shading) and cross-frequency phased coupled nested gamma. Potential actions are selected based on estimation of their relative expected value, with contrasting realized by coupled maps/graphs of the hippocampal system and vmPFC, with estimation and selection particularly influenced by the NAc and associated cortical systems. Lightly colored red hexagons indicate potential trajectories through (generalized) space, and dark red hexagons indicate chosen directions (either in imagination or reality). Imaginings cause increasing expectation (beta oscillations, red shading) for the value of potential actions, with corresponding accumulation of recurrent activity in body maps resulting in overt enaction once critical thresholds are surpassed. Abbreviations: Nac = nucleus accumbens; vmPFC = ventromedial prefrontal cortex; dmPFC = dorsomedial prefrontal cortex; SMA = supplementary motor area; Pre-SMA = presupplementary motor area; SEF = supplementary eye fields.

I suggest RPs—as motor predictions—are biophysically realized via accumulation of recurrent neural activity in frontal-parietal action-oriented proprioceptive body maps (Figure 6, Figure 7 and Figure 8), coupling with cingulate-insula salience networks, with patterns of enaction released when critical thresholds are surpassed in control hubs (e.g., pre-supplementary motor area) [354]. This threshold-crossing could be understood as an “ignition event” as described by global workspace theories [73,74,227,334], so constituting one (of multiple) means by which consciousness enters causal streams to leading to action. These periods of non-linear increases in activity may also correspond to periods where action-oriented body maps possess the highest degrees of integrated information, whose estimation could potentially correlate with measured strength of will [73]. Conscious intentions (as conjoined beliefs-desires) would contribute to ramping activity via the kinds of affectively-driven mental simulations described above [355,356] (Figure 5). This hypothesis of imaginative planning contributing to RPs is consistent with observed patterns of dopaminergic discharges, and also decreasing variance (and larger magnitude waveforms) leading up to volitional actions, indicative of value-based control processes [357,358].

Neurophenomenologically (Figure 7 and Figure 8), the feeling of “urge” preceding action corresponds to (non-linear) positive feedback interactions between frontal action hierarchies [351], posterior body-space-affordance hierarchies [94], and insulo-cingular (interoceptively-grounded) salience hierarchies [162] (Figure 6). These feelings are more than mere epiphenomena, influencing attentional selection for affective states, thereby modulating effective connectivity between these control hierarchies [154,160] (Figure 5). The stream of consciousness would further contribute to action selection via the counterfactual processing (e.g., simulated movements) and imaginative planning enabled by the hippocampal system [139,214,216,359] (Figure 4), including with respect to “Type 1” planned or “Type 2” capricious RPs [360]. These systems also contribute to imagining/predicting the consequences of more complex (and potentially meaningful) decisions [348], which would involve greater hierarchical depth and multiple realizability via particular actions from a “contrastive causation” perspective [361]. At these higher levels of abstraction, maximal explanatory power would be found in terms of more coarse-grained descriptions such as personhood and self-consciousness [162,207,264,315], so providing further neurocomputational grounding for agency. Finally, the “isolated qualia” model described above could also be relevant for explaining gaps between estimated ‘decision’ times and measured RPs, in that capricious actions will be more likely to be associated with quale-states that are difficult to take up into coherently introspectable streams of experience, yet nonetheless involve meaningful driving of dynamics by person-relevant values.

### 5.4. Qualia Explained?

Above we have considered prototypical qualitative aspects of experience, including pleasure, pain, and desire. Each of these “qualia” can be extremely rich in terms of their particular characteristics, underlying mechanisms, and functionalities, and the ways these vary across contexts. In what follows, I adopt a neurophenomenological approach [4,362,363,364] in beginning to explore how principles and mechanisms from FEP-AI can be used to cast light on how these aspects of our existence can be so fundamental, yet remain so mysterious.

#### 5.4.1. Emotions and Feelings

In attempting to analyze the nature of emotional experience, perhaps some of the continuing mystery is due to a lack of agreement on terminology. Damasio et al. [63,156,285,365], argue emotions can be ascribed to the value-oriented behavior of all living organisms, including single-celled organisms such as bacteria. However, Damasio reserves the word “feeling” for the conscious re-representation of emotions. Feldman-Barrett and LeDoux [179,284], in contrast, object to this more inclusive conceptualization of emotion, arguing instead that emotional language should be reserved for consciously-experienced affective states that are expressed and constructed through interpretive processes. LeDoux has even gone as far as to claim that emotions only arose via cultural evolution *after* the advent of language.

There are clear merits to both points of view. While less inclusive conceptualizations may avoid some confusions, they also miss opportunities to identify ways in which value and integrated informational dynamics are essential to all life [366,367]. I propose adopting an intermediate position, viewing emotions and feelings as respective action and perception components of action-perception cycles over large-scale changes in organismic modes. Relevant macroscale dynamics include diverse phenomena ranging from autonomic functions, to musculoskeletal body modifications [368], to nervous system alterations via neuromodulatory systems and effective connectivity from neural areas with high effective centrality (e.g., the amygdala complex). In addition to this cybernetic formulation of emotions as a kind of action, and feelings as a kind of perception, we may add an additional distinction as to the extent to which we are conscious of expressed emotions and sensed feelings. While potentially counter-intuitive, from this more inclusive point of view we may have both conscious and unconscious emotions, as well as conscious and unconscious feelings. This use of terminology would support many of the rationales for the positions described above, as well as many folk intuitions as expressed in normal linguistic use. If useful for communicative clarity and ethical considerations, additional distinctions can be made as to whether consciousness involves basic phenomenal awareness or is of a more complex access or autonoetic variety.

LeDoux [179,369] has argued that animals without complex language cannot be said to possess emotions, but merely have functional activity within “survival circuits.” These claims are justified by language providing necessary syntactic structures for the construction of complex extended self-models; or as LeDoux states: “No self, no fear”. This emphasis on the foundational importance of selfhood for conscious experience is largely compatible with the view presented here, and elsewhere [91]. Without extended self-processes, emotions and feelings will be qualitatively different than the kinds of emotions and feelings constructed by humans governed by (and governing) a symbolic order of being. However, within this cybernetic formulation, functional activity of “survival circuits” could contribute to the generation of emotions as large-scale organismic modes, yet still not be consciously expressed or felt.

Hence, all evolved cybernetic systems could be said to have emotions and feelings, but only systems capable of coherent integrative world modeling would consciously experience those affects [73,74]. These conscious systems likely include all mammals and birds, and possibly reptiles or fish if pallial tissue [370,371] is sufficiently elaborated to model system-world states with spatial, temporal, and causal coherence. Thus, we may take a middle way between the perspectives described above in viewing emotions and feelings as ubiquitous features of life, while simultaneously recognizing qualitative differences that emerge when these phenomena are associated with various kinds of consciousness. Both more and less inclusive conceptual stances are reasonable, but with respect to qualitatively different kinds of affective phenomena.

#### 5.4.2. What Is Value? Reward Prediction Errors and Self-Annihilating Free Energy Gradients

In FEP-AI, all living systems can be described as obeying a single objective of self-model-evidence maximization and prediction error minimization [372]. In this framework, organisms begin development by implicitly predicting the rectification of homeostatic (and later reproductive) prediction errors, so forming a foundation out of which all subsequent models grow. With experience, these modeling efforts come to apply to the modeling processes themselves and the experiences they generate, including models of what is likely to cause changes in prediction error. In this way, we come to predict ourselves minimizing prediction errors and experiencing associated mental states, including with respect to emotions and feelings. Through this associative chaining of memories from early organismic experiences, biological agents begin life being reinforced/punished as they continually attempt to engage in predictive homeostatic rectification (i.e., allostasis). However, organisms progressively learn sensorimotor contingencies for making these reward-related stimuli more/less likely to be available. Mechanistically, representations detecting these contingencies are themselves connected to midbrain value signals—e.g., orbitofrontal cortex 🡪 accumbens shell 🡪 ventral tegmental area 🡪 dopamine [110]—so allowing cortical models to drive reinforcement/punishment and shape adaptive policies for enaction.

This account has parallels with work on meta-reinforcement learning [342], where systems are initially given primary reward functions from which more capable secondary reward functions may be acquired from experience. From an FEP-AI perspective, these secondary predictions would constitute higher-order beliefs about likely patterns of prediction error minimization. According to candidate trace models [373], dopamine is likely to strengthen whatever predictions were most likely to contribute to its release by being most active leading up to phasic increases, so providing a partial solution to the credit assignment problem. If phasic dopamine increases are proportional to the rate of change of prediction error rectification [76,374,375,376,377], then the more quickly something minimizes prediction error, the more it will come to be predicted.

In these ways, organisms come to predict states of initial increases in prediction error, so that these free energy gradients (experienced as desire, or “wanting”) may be destroyed through enaction (experienced as pleasure, or “liking”) [378,379]. The creation and destruction of these gradients of anticipatory and consummatory reward will then stimulate dopamine release proportional to magnitudes of free energy minimization, as well as temporal intervals over which prediction errors are reduced [380]. These experiences, in turn, update beliefs and desires, whose counterfactual nature provide further sources of free energy to motivate future behavior. These mechanisms will shape organisms to predict themselves not only in homeostatic and reproductive states, but also diverging from these desirable modes of being, to the degree that such discrepancies between goals and actualities are anticipated to be manageable. Thus, through experience, we come to predict ourselves encountering initially negatively valanced states, which may become positively valanced when we annihilate these free energy gradients through either imagined or overt enaction, so establishing new goals/gradients to pursue/destroy in the future.

#### 5.4.3. Curiosity and Play/Joy

This prediction of prediction error minimization creates an interesting setup in which organisms end up being surprised when they do not find themselves riding down steep enough gradients of prediction error [76,374]. This is exactly what evolution would ‘want’ [17,381], since there is no limit to how evolutionarily fit an organism can be, and so organisms ought to always seek opportunities for realizing value in new ways. Driving of dopamine release by reward prediction errors may provide one means of realizing this evolutionary imperative for expansion in seeking opportunities for value realization. If the mechanisms underlying reinforcement and behavioral disinhibition are only activated for unexpectedly good outcomes, then organisms will always find themselves seeking to explore the limits of what they can attain. This exploratory impulse will be even stronger if accompanied by opportunities for refining models and satisfying curiosity-based desires, so realizing the intrinsic value of learning in addition to the extrinsic value of utility maximization [382,383,384,385,386].

Boredom, in contrast, represents a punishing process that functions in an inverse fashion to curiosity (and play). One mechanism for implementing this negative incentive could be found in predictive coding in terms of habituation. If organisms come to near-perfectly predict rewards—or consider associated stimuli to be not worth attending to—then this familiarity will result in prediction errors only being generated at lower levels of cortical hierarchies, which lack access to richly connected networks enabling conscious awareness [73]. Prediction errors failing to reach deeper levels will result in reduced recognition of features associated with those potential rewards. Both with respect to implicit predictions and explicit expectations, previously rewarding events that do not fully register will be experienced as disappointing in contrast to expected value [387,388], so resulting in stimulus-devaluation and reduced probabilities for selecting associated policies. Almost paradoxically, by becoming less (pleasantly) surprised by (or more familiar with) rewarding stimuli, organisms end up becoming more (unpleasantly) surprised relative to anticipated rewards, since predicted rewards never manifest in experience. Some evidence for this model can be found in over-rehearsed pleasurable acts being overly automatic, habitual, and progressively losing their hedonic tone. Between these twin masters of curiosity and boredom, agents are shaped to always expand their repertoire of policies for value realization, with growth continuing to the extent that these efforts are expected to result in increasingly desirable outcomes [386].

Under FEP-AI, we ought to expect living organisms—by virtue of being successful at existing—to be equipped with (or constituted by) system-defining prior expectations (or preferences) in which they are optimizing models of themselves doing the kinds of things which would be required for survival, including foraging for information. These modeling imperatives require organisms to enact system-world configurations dependent on policies with consequences in the future, and to also depend on policies not yet deployed [389]. This means successfully persisting adaptive systems must not only minimize free energy, but also expected free energy in (definitionally counterfactual) futures. A successful active inferential agent will expect itself to be maximizing information gain (i.e., precision-weighted prediction errors), while also avoiding the accumulation of cybernetic entropy [59,60,61,390,391] with respect to existential threats to the system. Within FEP-AI, this dilemma of balancing stability/plasticity tradeoffs is (boundedly optimally) resolved by gradient descent over a singular objective functional of expected free energy.

The maximal rate of reduction in overall expected free energy will be found in situations where agents are able to simultaneously balance imperatives for maximizing the intrinsic value of information/exploration with the extrinsic value of realizing preferred world states. This situation may be referred to as play, or “PLAY” [386,392,393] —potentially subjectively accompanied by “flow” states [394]—which, in maximizing reward, represents attracting states for organisms that places them precisely where they ought to be to maximize learning and evolutionary fitness [395]. The balanced conditions of play attract agents to a zone of proximal development [396]—or “edge of the adjacent possible” [397,398], and also the “edge of chaos” [239]—where learning rate is optimal, creating neither overly nor underly challenging conditions for promoting increasingly skillful engagement with the world [399,400]. 

These considerations help explain why we would not expect agents to minimize surprise by sequestering themselves in low-complexity environments. This is an a priori unlikely outcome, since such conditions would increase prediction errors from homeostatic regulatory nuclei and systems with which they (allostatically) couple. Further, such agents would both experience boredom and deprivation with respect to curiosity and play. Although we should also keep in mind that this supposed “Dark Room problem” [401] may not be completely solved by active inferential systems, as people often do seek out reduced complexity environments, whether due to the kinds of pathological beliefs associated with anxiety and depression [363,402], or by getting stuck at local maxima of excessive exploitation relative to exploration in model optimization.

#### 5.4.4. Synesthetic Affects

In the account described above, all affect is ultimately associatively linked to the rectification of either homeostatic or reproductive error signals, for which interoceptive consequences may be some of the most reliable sources of information [75]. However, these signals from the body’s internal milieu have poor spatial localizability and controllability. If spatiotemporal and causal contextualization are necessary for enabling coherent experience, then these constraints on sense-making could result in interoceptive information being attributed to non-interoceptive sources. The best available inference regarding these visceral (and vital) signals may be that they are both caused by and also inextricably part of the conditions with which they are associated. Theoretically, this could cause much of interoception to have a quasi-synesthetic quality, wherein poorly localizable signals become intimately entangled with (or ‘infused’ into) more easily modeled proprioceptive and exteroceptive phenomena (Figure 5). For example, we may both feel our body from within, while also projecting these feelings onto and into associated objects.

While it may seem odd to describe feelings as a kind of synesthesia, all perception may have at least some degree of synesthetic phenomenology by virtue of involving cross-modal blending [403,404,405,406,407]. Analogous (and likely overlapping) phenomena would include “oral referral” in which primarily olfactory percepts are mapped onto taste sensations [408]. Theoretically, synesthetic affects may provide a partial account of referred pain phenomena, in which damage to body parts are mistakenly attributed to another location [152,409]. To go out on a further speculative limb, the phenomenology of color perception may often be synesthetic in this way, with prototypical qualia such as the “redness of red” having its particular ‘textures’ due to interoceptive cross-mappings.

This *synesthetic-affects hypothesis* may have further support from descriptions of pleasure as a kind of “gloss” applied to objects of hedonic experience [378]. If accurate, this model could also explain part of why emotional experiences often have an ineffable quality when reported. That is, affects may heavily depend on information that is difficult to explicitly model, and for which modeling efforts usually involve a kind of anomalous inference that are personal feelings are inextricably—and essentially [410]—part of the conditions that evoke them.

Synesthetic affects may not only explain some of the ways that our feelings ‘color’ the world—for both good and ill—but also the phenomenology of will with respect to both motivation and effort (Figure 5, Figure 6, Figure 7 and Figure 8). In this view, the feeling of willing corresponds to a hybrid percept in which interoceptive states are mapped onto the effector systems by which intentions are realized. Thus, in addition to helping to explain otherwise mysterious aspects of experience, these synesthesia-like processes would also have extensive functional consequences. Perhaps most fundamentally, this kind of synesthetic phenomenology may help to establish senses of body ownership and minimal (embodied) selfhood upon which most aspects of mind ultimately depend. One line of evidence provided in support of these models is findings using augmented reality, in which superimposing interoceptive cardiac signals enhanced susceptibility to “rubber hand illusions” [411]. Intriguingly, such anomalous inferences are also moderated by tendencies for experiencing mirror-touch synesthesia and kinesthetic mirror illusions [197,412].

Predictive coding accounts of emotional active inference have been proposed in which prediction errors from interoceptive states can be minimized through either (a) changing autonomic conditions, or (b) changing related world states via mobilization of proprioceptive effector systems [75,413]. If synesthetic phenomenology increases the extent to which interoceptive states are tightly coupled with actions and perceived outcomes, then this conjunction would help establish affordance-informed salience mappings over perceptual contents, so facilitating action selection and planning [414]. As described above with respect to free energy flows across multimodal body maps and the generation of readiness potentials (Figure 5, Figure 6, Figure 7 and Figure 8), these tight perceptual couplings could strengthen patterns of effective connectivity between interoceptive and proprioceptive modalities. Such linkages would be more than mere epiphenomena, but would enable greater control energy from networks whose dynamics are ultimately grounded in evolutionary fitness and experiential histories with organismic value.

The subjective sense of presence [243] for affective phenomena may substantially depend on relatively tight associations between emotions and outcomes, so contributing to synesthetic mappings between feelings and inferred causes. If these links are disconnected—e.g., via insensitivity to interoceptive sensations or inabilities to imagine the realization of valued goals—synesthetic infusions of interoceptive value into other percepts would be compromised. In terms of consequences for normative functioning, severing synesthetic bridges to interoception could be involved in clinical conditions like anhedonia, alexithymia, the negative symptoms of schizophrenia, and even Cotard’s syndrome and Capgras illusions [154].

#### 5.4.5. The Computational Neurophenomenology of Desires/Pains as Free Energy Gradients That Become Pleasure through Self-Annihilation

Dopaminergic neuromodulation is commonly understood as indicating desire-related states [415,416], and also plays important roles in FEP-AI [417,418,419]. Dopamine modulates activity for representations of value-relevant stimuli, including actions associated with realizing valued goals. While dopaminergic functionality is complex [420], elevated signaling levels may be interpreted as indicating confidence that current policies/capabilities are likely to realize desired outcomes with respect to sensed or imagined stimuli. Relevant stimulus-features include both external reward cues as well as multimodal representations of activities involved in seeking valued goals, including avoiding undesirable outcomes.

In the predictive processing accounts of goal-oriented behavior described above, when an agent predicts itself obtaining value, but has not yet realized these desired outcomes, generated prediction errors correspond to discrepancies between representations for goal attainment, relative to estimated present or imagined likely states. These discrepancies are suggested to derive from iterative contrasting of desired and estimated likely states, occurring at theta frequencies orchestrated by hippocampal-prefrontal coupling [139,142] (Figure 4). As these comparison operations proceed, discrepant features generate increased activity as prediction errors, so drawing attention to and seeding imaginings with the most important features that need to be handled either by updating internal models or changing the world [105].

Much of the phenomenology of desire may represent the prediction of value-attainment, activating associated somatic and interoceptive concomitants of consummation, which are subjectively (and synesthetically) felt in body maps in places most associated with value realization (Figure 5). If these sensations are accompanied by temporary net decreases in predicting homeostatic or reproductive value-realization [76,374]—potentially mediated by opioid signaling [379,421]—overall unpleasant interoceptive inference may accompany these perceptions. In this way, the feeling of desire would be experienced as a kind of pain, with its particular characteristics depending on unique learning histories. However, painful desire can be transformed into pleasurable anticipation if we find ourselves predicting overall increases in value, so creating pleasurable contrasts with the discomfort of wanting. If the visceral concomitants of affective experiences become entangled with exteroceptive and proprioceptive percepts in the quasi-synesthetic fashion described above, then pleasure and pain (including desire) would be generated as interoceptive modes becoming infused into other modalities in particular ways based on historical associations.

To use a musical metaphor, in experiences of pain and unfulfilled desire, the overall melody is played in a more minor, or entropic [390,391] key/timbre. Alternatively, in experiences of pleasure and fulfilled desire—potentially including virtual fulfillment (i.e., pleasurable anticipation)—affective orchestras play melodies with greater consonance. One could view such soundtracks to the (fully immersive virtual reality) movies of experience as separate streams of information that help contextualize what is being seen on ‘screens’ over which we see stories unfold (Figure 6). However, it may be closer to experience to say that this metaphorical music enters into what we see and feel, imbuing (or synesthetically coloring) it with meanings. Indeed, we may be able to find most of the principles of affective phenomena to be well-reflected in our experiences of music [16,385,422], where we play with building and releasing tension, enjoying the rise and fall of more and less consonant (or less and more dissonant) melodies. In musical pleasure, we explore harmony and the contrast of disharmony, eventually expecting to return home to the wholeness of the tonic, but with abilities of our “experiencing selves” [423,424,425] to find satisfaction in the moment not necessarily being the reasons that our “remembering selves” find ourselves attracted to particular songs.

The affective melodies played by neural orchestras will be dominated by interoceptive modalities, the most ancient—both developmentally and evolutionarily speaking—and reliable indicators of homeostatic and reproductive potential [63,130,285,426,427]. Do we have relaxed and dynamic cardiac rhythms? Is our breathing easy or forced? Do we feel warm—but not too hot—or cold? Are our bowels irritated or copacetic? Do we feel full or empty inside? Do we feel like our body is whole and strong, ours to command where we will, if we wanted it? Or do we feel damaged and weak? This interoceptive information speaks to foundations of life and the cores of value out of which persons may grow.

#### 5.4.6. Desiring to Desire; Transforming Pain into Pleasure, and Back Again

How can we reconcile the experience of desire as a species of pain in light of the fact that we often desire to desire? While desiring may sometimes be desirable, it is not a pleasant thing to be in a state of unsatisfied hunger or thirst without believing this situation to be manageable. To be hungry or thirsty without cessation is to predict moving away from homeostasis and survival. Unless an organism can be confident that it will eventually rectify this situation, failing to satisfy such desires would indicate an existential threat to the system. Thus, we would expect desire unsatisfied to be experienced as a kind of pain. However, the pain of desire can then be transformed into pleasure—and back again (and so on)—by consummation, or the vivid imagination of attainment.

Can an agent ever come out ahead with this back and forth between pleasure and pain, either with respect its experiencing or remembering selves [424]? How can motivation be maintained if all pleasures will eventually be transformed back into kinds of pain through their absence? In addition to low-level mechanisms such as opioid signaling resulting in concomitant dopamine release [421], additional asymmetries between pleasurable and painful experiences may be found in predictive coding mechanisms. That is, more changeable patterns will be more likely to violate expectations—by virtue of being difficult to precisely track—and so experiencing/remembering will likely be dominated by transitions between pleasure and pain, especially if accompanied by precipitous or punctuated alterations [76,248,374,380,428]. If seeking without finding results in relatively gradual accumulation of desire, and if consummation tends to rectify situations more rapidly, then experience and memory for successfully enacted goals will have an overall pleasurable (and reinforcing) quality. Additionally, by virtue of being substantially generated by an agent’s own (potentially intentional) actions, the greater predictability of consummatory acts might allow attentional resources to be marshalled in ways that allow for more extended conscious processing of pleasurable experiences. Finally, some symmetry breaking with respect to pain and pleasure may come from the motoric nature of attention described above, in that the experience of attending to pleasurable experiences will be more likely to be reinforcing both in the (extended) moment as well as across time. [Note: The conditioning of top-down attention also suggests that some quasi-psychodynamic phenomena are to be expected as almost inevitable consequences of the laws of learning.] However, this pleasure is not something that natural selection ‘wanted’ us to have and hold onto, but to be continually “SEEKING” [392], thereby maximizing fitness.

#### 5.4.7. Why Conscious Feelings?

Consciously-experienced feelings may provide unified attractors for coordinating global organismic states [130,285]. While emotional shaping may occur without consciousness, these affects may be more likely to entrain the overall system when integrated into coherent fields of experience. Even if these feelings take the form of “isolated qualia” without conscious access (as described above), these self-stabilizing cores may still provide sources of greatly elevated control energy. However, this entraining power would be even greater when made consciously accessible in ways that afford planning and continuous adjustments of actions based on organismic value. This mapping of hedonic states onto consciously introspectable models of enaction also provides a partial means of handling the credit assignment problem, via conjoining value and actions in both experience and memory. If affects took place “in the dark” without feeling like anything, they would be unable to strongly influence events, nor be coherently integrated into explicit modeling and planning, including plans involving pursuing those feelings as ends in and of themselves, such as in the domains of play and art.

### 5.5. Facing up to the Meta-Problem of Consciousness

The hard problem of consciousness asks, how can it be that there is “something that it is like” to be a physical system [429,430]? The “meta-problem” of consciousness refers to the (potentially more tractable) challenge of addressing why opinions and intuitions vary greatly with respect to what could meaningfully answer this question [431]. As suggested elsewhere [73], one potential solution to the meta-problem may derive from the unavailability of bridging principles, which would cause prospects for explaining consciousness to seem either impossible (perhaps even in principle), or merely (extremely) difficult. An additional solution to the meta-problem may be found in the nature of explanations for various qualia: while perhaps intuitive after consideration, at first glance some of the models proposed above seem to directly contradict experience, such as desire constituting a species of pain, and vice versa [152]. Other explanations of aspects of experience may not necessarily contradict intuitions, yet may nonetheless seem irreducibly strange and so prima facie implausible, such as the model of synesthetic affects described above. However, if it is indeed the case that some of the most fundamental and familiar aspects of experience are difficult to recognize upon close inspection, then this is itself something in need of explanation.

Much of this seeming paradox of the unrecognizability of the most fundamental and familiar could be resolved if such aspects of experience are likely to become “phenomenally transparent” [49,432], and so resistant to introspection. Neurocomputationally, the contents of perception in any given moment are likely entailed by synchronous beta complexes with particular zones of integration [73,74], but with these local inferences requiring further integration into larger (alpha- and theta-synchronized) complexes for phenomenal and access consciousness. Such broader integration may not end up occurring if predictive coding mechanisms are so successful that they are capable of “explaining away” aspects of experience before they can be consciously registered. That is, iterative Bayesian model selection unfolds over multiple (potentially nested) levels of hierarchical depth, and so if explaining away observations is largely successful via smaller beta complexes closer to the modalities, that information would never reach more richly connected cores/subnetworks enabling coherent world modeling and experienceable perception. Alternatively, such information could give rise to experience in the form of “isolated qualia,” yet fail to achieve conscious accessibility due to the transient nature of these quale states. In these ways, that which is most fundamental and familiar would almost inevitably become nearly invisible to introspective access.

The meta-problem may be as conceptually rich as the Hard problem itself. Further promising approaches may involve paradoxes from functional “Strange loops” and self-reference [433], computational limits of recursion [434], and seeming paradoxes deriving from mechanisms by which egocentric perspective is established [181]. Finally, some solutions may be sociological in nature, potentially reflecting a legacy of “physics envy” in the mind sciences [435]. Not only have we lacked bridging principles and understanding of embodiment as the core of selfhood and experience, but scientific practice both implicitly and explicitly denigrated subjectivity after the decline of introspectionism and rise of behaviorism. Given this taboo on subjectivity—i.e., the very thing we would hope to explain with respect to consciousness—why should we have been surprised if we lacked satisfying understanding of the nature(s) of experience? Finally, some of the (Hard) problem may derive from frames in cognitive science that rendered all Cartesian framings of mental functioning taboo. That is, if quasi-Cartesian intuitions were actually semi-faithful representations of the nature(s) of mind and brain, then why should we be surprised if our scholarship—and its denigration of folk psychology [436]—failed to provide satisfying accounts of the nature(s) of our conscious agency?

## 6. Conclusions

*“The intentionality of all such talk of signals and commands reminds us that rationality is being taken for granted, and in this way shows us where a theory is incomplete. It is this feature that, to my mind, puts a premium on the yet unfinished task of devising a rigorous definition of intentionality, for if we can lay claim to a purely formal criterion of intentional discourse, we will have what amounts to a medium of exchange for assessing theories of behavior. Intentionality abstracts from the inessential details of the various forms intelligence-loans can take (e.g., signal-readers, volition-emitters, librarians in the corridors of memory, egos and superegos) and serves as a reliable means of detecting exactly where a theory is in the red relative to the task of explaining intelligence; wherever a theory relies on a formulation bearing the logical marks of intentionality, there a little man is concealed.”*

—Daniel Dennett [1]

These explorations have attempted to repay as many “intelligence loans” as possible by creating an embodied backing for intentionality, so providing a common currency for understanding cognition. I have suggested that consciousness is generated from dynamic predictive cores, centered on embodied self-models, functioning as cybernetic controllers for agents embedded in environments within which they seek valued goals. To realize these values, agents engage in imaginative planning in which they chain inferences from desired outcomes back to present-estimated states, with enaction realized via multilevel action-oriented body maps. For these quasi-homunculi, intentional control is driven by beliefs and desires, understood as free energy gradients, which are annihilated when prediction errors are minimized through skillful enaction, so establishing new goals to pursue in the future. Through a variety of simulated actions, embodied self-models both influence and are influenced by high-level representations from interoceptive and exteroceptive inferential hierarchies, so providing bases for various forms of conscious access, metacognition, and self-knowledge. This deeply embodied architecture provides enactive bases for most mental operations discussed in cognitive science, including means by which conscious mental states can causally influence attention, working memory, imagination, and action. In these ways and more, understanding the radically embodied foundations of conscious minds may vindicate much of folk psychological and traditional conceptions of selves containing both multiplicity and unity, and of will defined by both constraints and freedom.

## Figures and Tables

**Table 1:** Example of goal-oriented behavior via iterated comparisons between imagined (dark grey) and estimated (light grey) states.

Examples of Imaginative Policy Selection			
Counterfactual Predictions	Observations	Prediction-Errors and Associated Memories	Types of Value
…Drinking tea	Not drinking tea	Body states associated with drinking	Pragmatic
Finding tea in cup	Not seeing tea	Surprise and reorienting	Epistemic
Making tea	Sitting at desk	Location and object affordances	Pragmatic
Going to kitchen	Sitting at desk	Location and locomotion	Pragmatic
Effort of standing	Standing	Motion and accompanying visceral sensations	Pragmatic
Drinking tea	Not drinking tea (but closer)	Body states associated with drinking	Pragmatic
Making tea	Locomoting to kitchen	Location and object affordances	Pragmatic
Holding tea bags	Standing in kitchen	Location, position, and object affordances	Pragmatic
Finding tea bags	Scanning kitchen	Surprise and re-orienting	Epistemic
Drinking tea	Not drinking tea (but closer)	Body states associated with drinking	Pragmatic
Steeping tea	Pouring water	Location, position, and object affordances	Pragmatic…
…Drinking tea	Holding hot cup	Body position	Pragmatic
Burning mouth	Holding hot cup	Body states associated body damage	Pragmatic
Sipping slowly	Not burning mouth	Body states associated with drinking	Pragmatic

**Table 2:** Kinds of attentional biasing via partially-expressed motor predictions.

A Taxonomy of Attending via Partially-Expressed Motor Commands	
Kinds of Attention	Relevant Actions
Spatial biasing	(a) Foveation (b) Head or trunk turning/orienting (c) Pointing (d) Other directional gestures (e) Locomotion
Feature and object focusing	(a) Speech or sound production (i.e., phonological loops) (b) Actions related to particular morphological, locational, emotional, or affordance characteristics (i.e., physically interacting-with or constructing) (c) Patterns of motion typically associated with particular objects (d) Physical sketching (e) Physical interaction with or locomotion through some (potentially synesthetic) memory-palace-like mapping
Following temporal patterns	(a) Rhythmic speech or sound production (b) Rhythmic motions of gross musculature (c) Rhythmic motions of sensory apparatuses (e.g. foveations, auricular constrictions, etc.)
Duration-based attending	(a) Extended production and tracking of accumulation of simulated rhythms (i.e., inner clocks) (b) Enacting events/processes with temporal extent without being clearly rhythmic (c) Mapping time onto a spatial reference frame (i.e., spatialization of time)

## Data Availability

This manuscript does not have associated data.
